# The Genetic Architecture of Congenital Diarrhea and Enteropathy

**DOI:** 10.1056/NEJMoa2405333

**Published:** 2025-04-03

**Authors:** Zeenat Gaibee, Neil Warner, Katlynn Buda Gwilt, Wenjuan Li, Rei Guan, Michael Yourshaw, Ryder Whittaker Hawkins, Christiane Zorbas, Jonathan St. Germain, Mahdi Tabatabaie, Suli Mao, Vered Pinsk, Baruch Yerushalmi, Lee-kai Wang, Stanley F Nelson, Laura Wozniak, Dror S Shouval, Manar Matar, Amit Assa, Nathaniel Frost, Lissette Jimenez, Sari Acra, Thomas Walters, Stephen Mouat, Michael Li, Denis LJ Lafontaine, Matthew Tyska, Brian Raught, Yaron Avitzur, Wayne I Lencer, James R Goldenring, Martín G Martín, Jay R Thiagarajah, Aleixo M Muise

**Affiliations:** 1 Division of Gastroenterology, Hepatology, and Nutrition, The Hospital for Sick Children, Toronto, Ontario, Canada.; 2 Cell Biology Program, Research Institute, Hospital for Sick Children, Toronto, Ontario, Canada.; 3 Division of Gastroenterology, Hepatology and Nutrition, Boston Children’s Hospital; Harvard Medical School, Boston, MA.; 4 Department of Human Genetics, University of California, Los Angeles, Los Angeles, CA, USA.; 5 RNA Molecular Biology, Fonds de la Recherche Scientifique (F.R.S./FNRS), Université libre de Bruxelles (ULB), Biopark campus, B-6041 Gosselies, Belgium.; 6 Princess Margaret Cancer Centre, University Health Network, and Department of Medical Biophysics, University of Toronto, Toronto, ON, Canada, Department of Medical Biophysics, University of Toronto, Toronto, ON, Canada.; 7 Department of Cell and Developmental Biology, Epithelial Biology Center, Vanderbilt University School of Medicine, Nashville, TN, USA.; 8 Division of Pediatrics, Pediatric Gastroenterology Unit, Soroka University Medical Center and Faculty of Health Sciences, Ben-Gurion University of the Negev, Beer-Sheva, Israel.; 9 Department of Pediatrics, Cedars-Sinai Medical Center, Los Angeles, CA, USA; 10 Institute of Gastroenterology, Nutrition and Liver Diseases, Schneider Children’s Medical Center of Israel, Petach Tikva, affiliated with the Faculty of Medicine, Tel-Aviv University, Tel Aviv, Israel.; 11 The Juliet Keidan institute of Pediatric Gastroenterology and Nutrition, Shaare Zedek Medical Center, The Hebrew University, Jerusalem, Israel.; 12 Division of Pediatric Gastroenterology, Hepatology and Nutrition, Vanderbilt University Medical Center, Nashville, TN.; 13 Department of Paediatric Gastroenterology and Hepatology, Starship Children’s Health, Te Toka Tumai, Auckland, New Zealand.; 14 Center for Computational Medicine, Research Institute, Hospital for Sick Children, Toronto, Ontario, Canada.; 15 Department of Pediatrics, Division of Gastroenterology and Nutrition, Eli and Edythe Broad Center of Regenerative Medicine, Mattel Children’s Hospital and the David Geffen School of Medicine, University of California Los Angeles, Los Angeles, CA.; 16 Department of Paediatrics and Biochemistry, University of Toronto, Toronto, Ontario, Canada

## Abstract

**Background:**

Next-generation sequencing (NGS) has enabled precision therapeutic approaches that have improved the lives of children with rare diseases. Congenital diarrhea and enteropathies (CODE) have high morbidity and mortality. While CODE treatment is largely supportive, emerging targeted therapies based on genetic diagnoses include specific diets, pharmacologic treatments, and surgical interventions.

**Methods:**

We analyzed the exomes or genomes of infants with suspected monogenic congenital diarrheal disorders. Using cell and zebrafish models, we tested the effects of variants in newly-implicated genes.

**Results:**

In our case series of 129 infants with suspected monogenic congenital diarrheal disorders we identified causal variants, including a new founder *NEUROG3* variant, in 62 (48%) cases. Using cell and zebrafish models, we also uncovered and functionally characterized three novel CODE genes, *GRWD1*, *MYO1A*, and *MON1A*.

**Conclusion:**

We have characterized the broad genetic architecture of CODE disorders in a large case series of patients and identified three novel CODE disorders.

Congenital diarrhea and enteropathies (CODE) are a group of rare disorders that primarily affect intestinal epithelial cell function, leading to infantile onset diarrhea and poor growth. CODE molecular defects can be classified into 5 categories: epithelial nutrient/electrolyte transport, enzymes/metabolism, trafficking/polarity, endocrine cell dysfunction, and immune dysregulation–associated enteropathy^1,2^. CODE has substantial morbidity and mortality. Patients often require lifelong fluid and nutritional management.^3^ Genetic causes include pathogenic variants in *MYO5B* (Microvillus Inclusion Disease (MVID)),^4^ *EPCAM* (Tufting enteropathy^5^), *NEUROG3* (enteric anendocrinosis^6^), *DGAT1* (protein-losing enteropathy^7^) and *SLC9A3* (congenital sodium diarrhea^8^). Treatment options are currently limited. However, understanding some of the genetic causes of CODE has led to targeted therapies such as dietary treatments^3^ and the development of preclinical pharmacological treatments^2^, such as leflunomide^9^ for the treatment of intestinal epithelial defects observed in TTC7A defiency^10^. Studies of monogenic inflammatory bowel disease^11^, and small single-center diarrheal disorder case series^12,13^ have examined 1005, and 6 and 24 patients respectively. Here, we report a multi-center study to systematically examine genes underpinning CODE disorders.

## Methods

### Patient Population

Infants had a clinical diagnosis of congenital diarrhea (defined as chronic diarrhea lasting more than 2 weeks and starting in infancy with no attributed anatomical, infectious or allergic cause. (See ref. 1 for specific types of diarrhea based on stool osmolality including osmotic, secretory and mixed.) They were treated locally at the Hospital for Sick Children (HSC) in Toronto, Canada, Vanderbilt University Medical Center (VUMC), Boston Children’s Hospital (BCH), and the University of California Los Angeles (UCLA), and referred from across the United States to VUMC, BCH, and UCLA, and from several international institutions to BCH and UCLA. Families who previously consented to genetic testing and further research studies were analyzed as part of the PediCODE consortium (Pediatric Congenital Diarrhea and Enteropathies; www.PediCoDE.org) using a single NIH protocol IRB-P00027983 for UCLA, BCH, and VUMC in the United States and REB 1000072542 for the HSC in Canada. All families provided written consent to participate in the study; at least one parent or guardian provided written consent for the participation of their children. Demographic characteristics of the study population are provided in Table S1. Individual CODE patients or families were previously sequenced, and raw reads were aligned as described in Supplemental Material. Principal component analysis of the case series demonstrated a well-distributed ancestral diversity (Figure S1). Quality-control measures are outlined in the Supplementary Materials and Tables S2–6.

**Figure 1.**
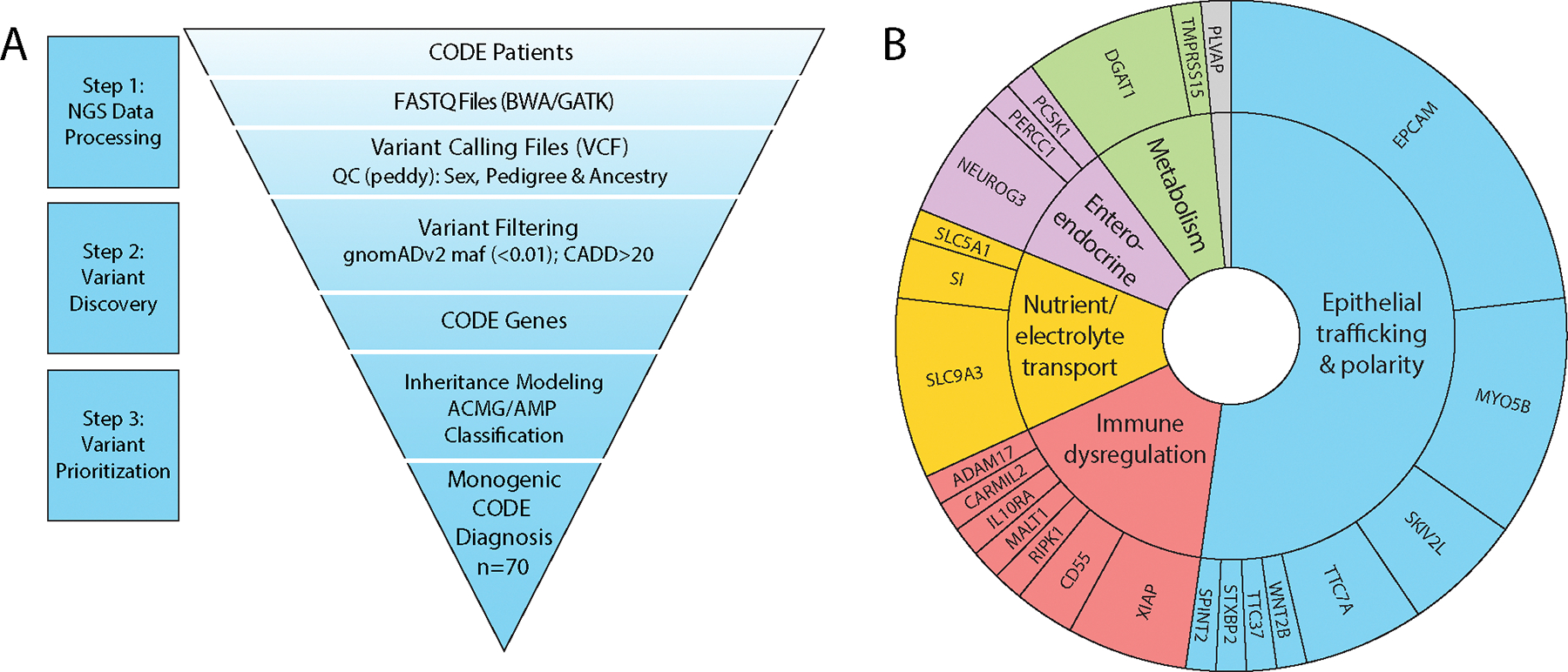
The Genetic Architecture of CODE. **A.** Patient flow diagram showing DNA sequencing and single bioinformatics pipeline and variant annotation with quality control processes. **B.** CODE genes identified in PediCODE Consortium patients.

An overview of the genetic analysis of the case series and identification of monogenic CODE genes is provided in Figure 1A. Briefly, GEMINI (short for “genome mining”) was used to identify candidate variants based on known inheritance models of previously identified genes that, when variant, are known to cause CODE and are listed in OMIM^14^ (Table S7). We first searched exome data for rare (gnomAD^15^ allele frequency < 0.01) and damaging (Combined Annotation Dependent Depletion (CADD) Score^16^ > 20) variants in previously published CODE genes. We then carried out manual filtering based on confirmatory inheritance pattern, segregation, previous annotation in ClinVar, concurrence with clinical features associated with phenotypes of known genetic disease, and overall pathogenicity based on the ACMG/AMP classification^17^. An additional manual screen of the remaining cases was carried out to determine prior ClinVar-annotated pathogenesis and to search for novel CODE genes. Cases were considered solved when an ACMG/AMP pathogenic or likely pathogenic classification was identified in an OMIM CODE gene that fit the case description and expected inheritance pattern.

Functional studies to investigate novel candidate genes included the use of proximity-dependent biotin identification (BioID), cell-based assays, and the creation of novel zebrafish models (see methods in the Supplementary Appendix). Briefly, BioID was used to identify alterations in interactions between proteins encoded by candidate CODE gene variants, as compared to their wild-type counterparts with known binding-partner proteins. High-confidence interactors were defined as those with a Bayesian false discovery rate (BFDR) ≤0.01. Zebrafish studies were carried out at the Zebrafish Genetics and Disease Models Facility at The Hospital for Sick Children, Toronto, Canada, with approval from the Animal Care Committee, Animal Use Protocol #65759. Candidate genes in zebrafish were knocked out using a CRISPR-Cas9 system (Figure S2 and S3). The Student’s t-test was used to compare the means of two groups of zebrafish. RNA was extracted and sequenced from pooled wildtype or variant zebrafish and differential expression analysis was carried out using the DESeq2 package in R. Several cell lines, including CACO-2_BBE_ (monolayers), Hela, MDCK-FCRN, and HT-29 cells were used for functional studies; statistical analyses of these studies are described in the figure legends and Supplementary Appendix.

## Results

### Genetic Architecture of CODE

We analyzed NGS data obtained from the PediCODE consortium sites (PediCODE.org). Overall, 139 infants (including 10 sibling pairs) who presented with CODE, and 182 parents and siblings without CODE were analyzed (Table S1). Of the 129 families analyzed, 98 had at least one family member (other than the proband) exome sequenced to assist with making a genetic diagnosis (Tables S2). Fourteen probands (1 sibling pairs) originated from consanguineous matings, as suggested by a relationship coefficient exceeding 0.1 (Table S3). In 62 of 129 probands, we identified causal variants in one of 24 known monogenic CODE genes (Figure 1B, Table S8). Of these 62 diagnosed probands, 58 (94%) had autosomal recessive (AR) and 4 (6%) had X-linked disorders (Table S8). Cases in nearly half the probands were linked to genes involved in epithelial trafficking and polarity, including *EPCAM* (12 cases), *MYO5B* (8 cases), *SKIV2L* (3 cases), and *TTC7A* (4 cases). Other CODE gene variants detected in this analysis included those in *SLC9A3* (5 cases), DGAT*1* (5 cases), *XIAP* (4 cases), and *NEUROG3* (3 cases). Eight of the 10 sibling pairs who presented with CODE harbored the same pathogenic variant as the proband (Figure 1B and Table S8). Collectively, variants in these 8 genes caused disease in 52 (74)% of the identified cases. Seven infants from 6 unrelated families with Tufting Enteropathy carried a previously reported founder variant identified in the Arabic Gulf, *EPCAM* Q167Pfs*21^18^. Four infants with enteric anendocrinosis from 3 unrelated Bedouin families (each with a WES calculated relationship coefficient < 0.1) had a novel variant in NEUROG3 variant (Q137R).

Three probands had variants that were not discoverable through exome sequencing; genome sequencing or Sanger sequencing was required to identify the causal variant in these probands, including Proband 68, who had an *XIAP* deletion. WGS also identified a *PERCC1* variant in Proband 57, a recently identified gene required for enteroendocrine cell function that was previously unannotated and, therefore, not captured by WES^19^. Proband 5 had an intronic splice donor site variant in *SLC9A3* that was missed due to poor coverage of Exon 8 (Figure S4). Furthermore, examination of median sequencing depth showed that several genes had poor coverage of exon 1, as has been noted in previous studies. Notably, several exons in *MYO5B*, the second most common cause (when pathogenically variant) of CODE in this study, had poor sequence, suggesting that deeper sequencing of *MYO5B* should be considered if there is a high suspicion for microvillus inclusion disease and negative results from *MYO5B* exon sequencing (Figure S4).

Further interrogation of the exome data from patients with negative WES analysis revealed three novel candidate CODE genes based on genetic heritability, population frequency, CADD score, known gene function, or available animal models (detailed in Table S9).

### Genetic and Functional Significance of *GRWD1* Variants

The first candidate gene, *GRWD1,* was identified in a sib pair (comprising a male and female) who presented with congenital diarrhea requiring parenteral nutrition and diffuse arterial hypoplasia. Exome sequence analysis revealed rare and damaging maternally inherited 19:48451128 A/G (p.H307R; CADD score 25) and paternally inherited 19:48452786 G/T (p.V368F; CADD score 25) *GRWD1* variants.

**Figure 2.**
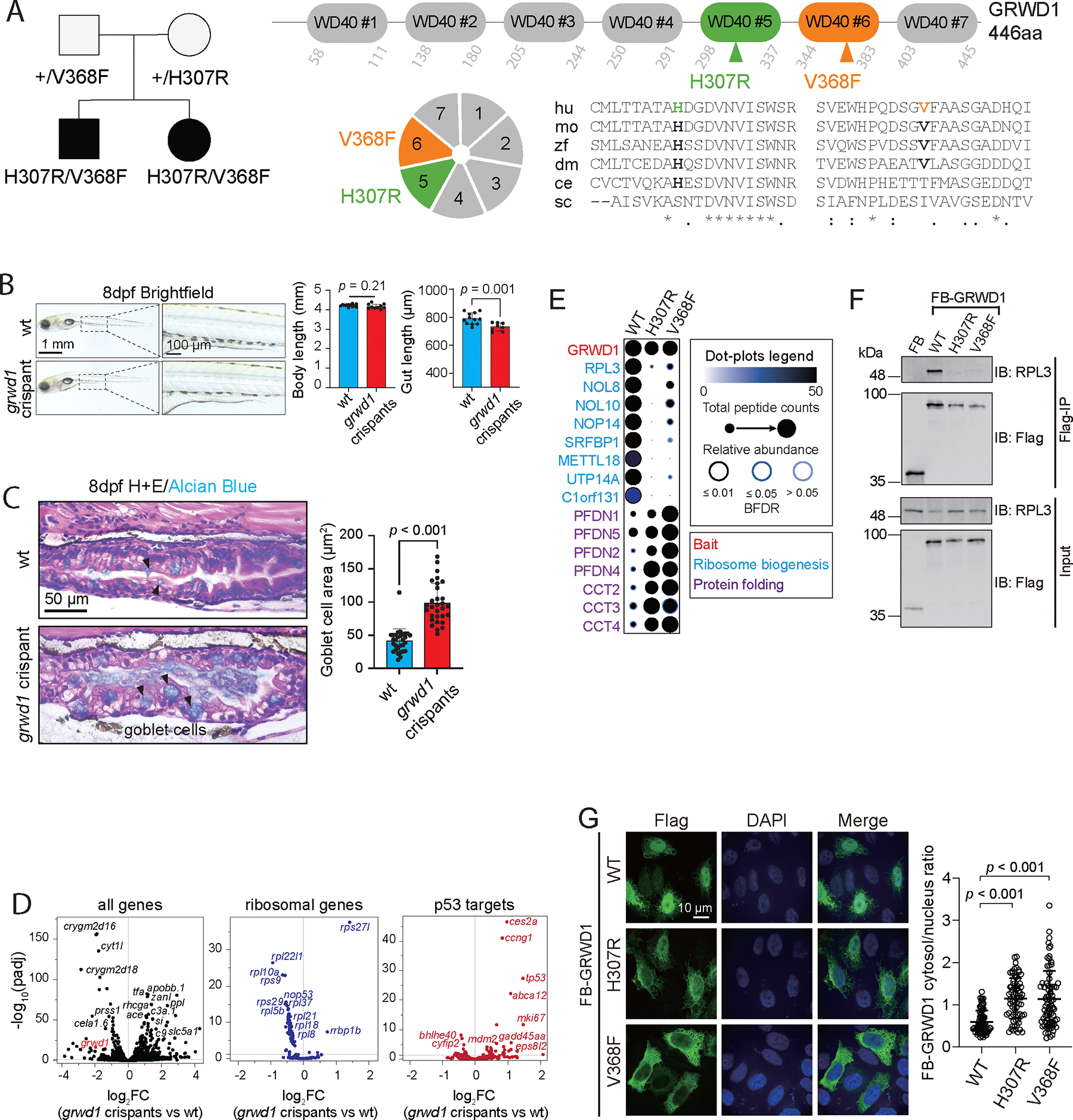
Genetic and Functional Studies of GRWD1 Variants. **A.** Trio WES identified biallelic variants in *GRWD1* in two CODE probands. Pedigree is on the left, and cartoon depiction of the seven WD40 repeats of GRWD1 and amino acid conservation across species of the 2 variants identified in 2 CODE probands (hu, human; mo, mouse; zf, zebrafish; dm, *Drosophila melanogaster*; ce, *Caenorhabditis elegans*; sc, *Saccharomyces cerevisiae*). Alignments generated by clustalo; (*) conserved residues; (:) strongly similar residues; (.) weakly similar residues. **B.** Representative bright-field images of wt (N = 12) and *grwd1* crispants (N = 10). Body length and gut length were quantitated by Fiji. Statistical differences were determined by Student’s t-test. Mean ± SD. **C.** Gut morphology of wt and *grwd1* zebrafish crispants: Representative images of wt and *grwd1* crispant zebrafish at 8 dpf with H&E and alcian blue staining, N = 6. The area of each goblet cell was measured using the Fiji selection tool to draw region of interest (N = 30). Statistical differences were determined by Student’s t-test. Mean ± SD. **D.** Volcano plot comparing RNA-sequencing data between wt and *grwd1* crispants at 8 dpf (N = 15 pooled larvae/group, duplicated, GRCz11 annotation, DESeq2 Wald, adjusted p = 0.01). All genes and subsets of differentially expressed ribosomal protein and p53-signaling genes are displayed. **E.** ProHits-viz Dot Plot diagram depicting BioID data for select proximity interactors of the WT and CODE variant GRWD1 proteins. Dot size indicates relative abundance of each interacting partner detected across each of the three “bait” proteins. Dot shade indicates total peptide counts detected for each interactor, and the shade of the ring surrounding each dot (blue to black) indicates the confidence level (Bayesian False Discovery Rate) of each indicated bait-prey interaction. Interactors grouped by functional annotation, as indicated. For statistical analysis, a Bayesian FDR was assigned to identified proteins using SAINT (v3.6.1; 20 BirA*Flag-only controls compressed to 4). **F.** HEK293 T cells were transfected with either Flag-BirA* alone or a Flag-BirA* tagged GRWD1 protein (WT or CODE variant, as indicated). Flag-tagged proteins were precipitated with Flag Agarose Affinity Gel and eluates analyzed by western blotting, using antibodies directed against the Flag epitope or the RPL3 protein. Data are representative of 3 independent experiments. **G.** Hela cells expressing Flag-BirA*- GRWD1 WT, H307R, or V368F proteins were fixed and stained for Flag epitope (green) and DAPI (blue). Scale bar 10μm. GRWD1 cytosol/nucleus signal ratio was calculated using Volocity. Statistical differences were determined by Student’s t-test. Error bars represent SD from 3 independent experiments, with >70 cells analyzed.

Glutamate-rich WD40 repeat containing 1, GRWD1, is a highly conserved member of the WD40 protein family (Figure 2A). It is required for late steps of large ribosomal subunit assembly^20^ and is a regulator of p53^21^. As knockout of *grwd1* was lethal in zebrafish, we generated first-generation (F0) mosaic *grwd1* mutant zebrafish using CRISPR-Cas9 editing (crispant *grwd1*) that survived until 15 days post fertilization (dpf; Figure S2). Though crispant *grwd1* larvae at 8 dpf had normal body length, they displayed significantly reduced gut length (Figure 2B) with disrupted intestinal architecture including enlarged, rounded goblet cells, disorganized enterocytes, and irregular gut lumen surface compared to the intestines of wildtype control fish (Figure 2C). The goblet cell area was greater in crispant *grwd1* zebrafish than in control fish (Figure 2C). Differential expression analysis and pathway analysis of whole 8 dpf *grwd1* crispant larvae revealed perturbation in several ribosomal and p53 regulated genes (Figure 2D).

Ribosome biogenesis dysfunction triggers nucleolar surveillance^22^, a regulatory loop in which unassembled ribosomal proteins forms a complex with the ubiquitin E3 ligase Hdm2, which in turn leads to p53 stabilization and increased expression of the target genes of p53^23^. Consistent with the activation of nucleolar surveillance, differential RNA expression analysis of whole 8 dpf *grwd1* crispant larvae revealed perturbation in several ribosomal and p53 regulated genes (Figure 2D). To characterize the GRWD1 CODE protein variants, we conducted proximity-dependent biotin identification (BioID; Figure 2E and Table S10)^24^ on the GRWD1 protein and the missense variants H307R and V368F in human HEK293 Flp-In T-REx cells. BioID of GRWD1 identified a high-confidence interaction with a single ribosomal protein, RPL3, and with multiple ribosome biogenesis factors important for nucleolar steps of large subunit assembly^25^. Both H307R and V368F variant proteins displayed reduced interactions with RPL3 and ribosome assembly factors and increased interactions with prefoldin and CCT family protein folding chaperones. In budding yeast and *C. elegans*, the GRWD1 orthologues rrb1 (yeast) and GRWD-1 (*C. elegans*) are reported to act as chaperones for rpl3^26,27^, and thus critical to the formation and function of the ribosomal peptidyl transferase center^28^. Decreased RPL3 binding for the GRWD1 CODE variants was confirmed in an orthogonal affinity purification assay (Figure 2F). Finally, Flag-tagged versions of the H307R and V368F GRWD1 variants were predominantly localized to the cytoplasm and the wildtype protein was primarily localized to the nucleus in transfected HeLa cells (Figure 2G). The GRWD1-variant proteins were mislocalized and had defective RPL3 chaperone activity, resulting in defective ribosomal biogenesis; and our *grwd1* crispant zebrafish model demonstrated intestinal goblet cell abnormalities. We conclude that *GRWD1* deficiency results in defective ribosomal biogenesis, and specifically in the gut, goblet cell dysfunction resulting in a novel CODE.

### Genetic and Functional Significance of *MYO1A* Variants

**Figure 3.**
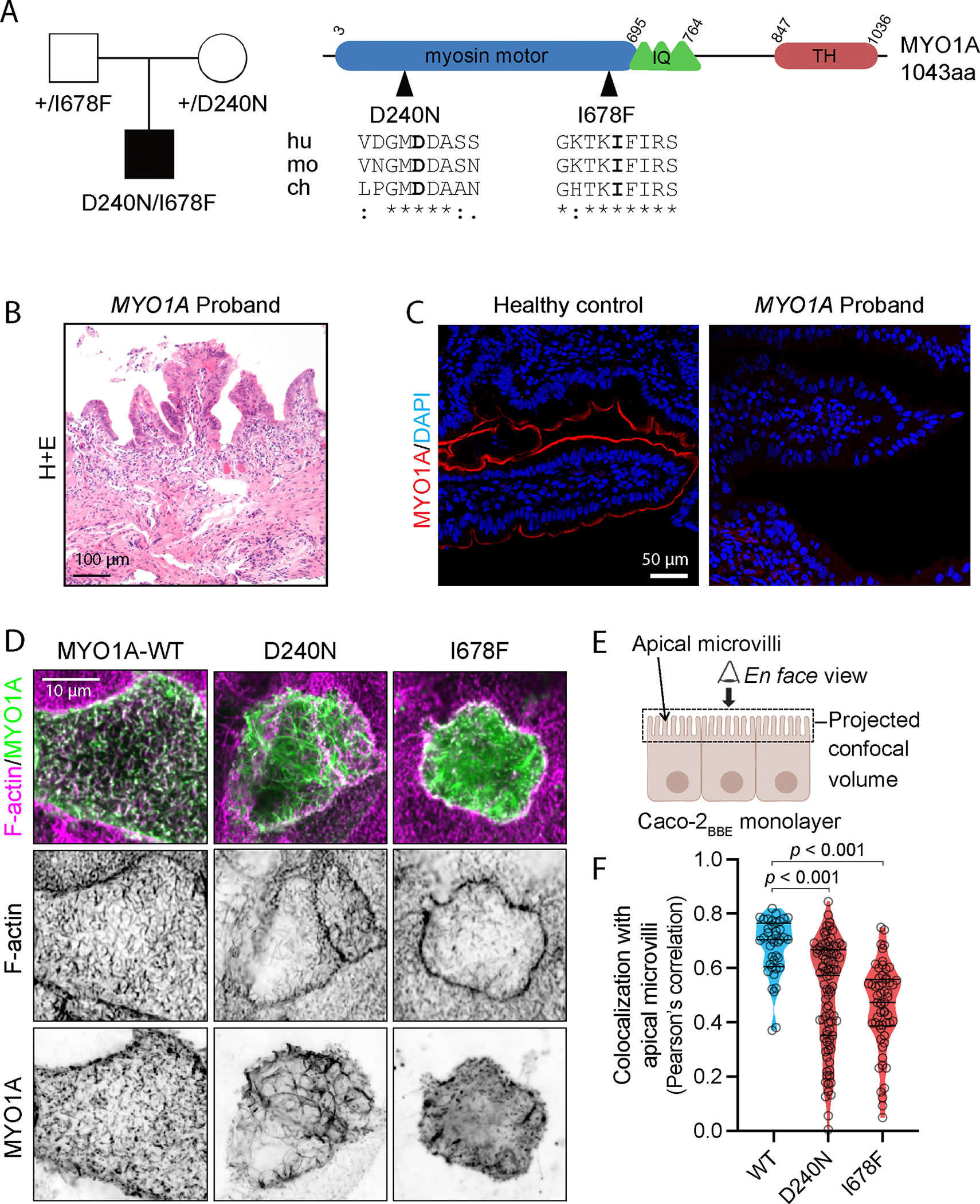
Genetic and Functional Studies of MYO1A Variants. **A.** Trio WES identified biallelic variants in *MYO1A* in a CODE proband. Pedigree is on the left, and a schematic illustration of the protein domain architecture is on the right, highlighting the amino acid change and its conservation across species: human (hu), mouse (mo), zebrafish (zf), and chicken (ch). IQ = IQ Motif, TH = Tail Homology. **B.** Hematoxylin and eosin staining of intestinal biopsy from the proband. **C.** Immunofluorescence images for healthy control and MYO1A-I678F proband tissue showing MYO1A (red) and DAPI (blue). **D.** Maximum intensity projections of confocal volumes showing localization of wild-type (WT), D240N, and I678F variants of EGFP-MYO1A (green) in CACO-2_BBE_ cells, fixed and stained with phalloidin to highlight F-actin (magenta). Top row shows two-channel merge images; inverted single channel images for EGFP and phalloidin are shown beneath each merge. Scale bars, 30 μm **E.** Cartoon depicting orientation of the image planes shown in D relative to the CACO-2_BBE_ monolayer and the volume sampled during confocal imaging. **F.** Pearson’s correlation coefficients calculated between green (MYO1A construct) and magenta (F-actin) channels on a per-cell basis; this value reflects the extent of colocalization between each expressed protein and the microvillar actin cytoskeleton. Each point represents a measurement from a single cell; N = 40, 87, 58 cells for WT, D240N, and I678F, respectively. Solid black lines denote median correlation coefficients. Statistical differences were determined by Kruskal-Wallis ANOVA test.

The second candidate gene, *MYO1A*, was identified in a male infant who presented at two months of age with an abrupt onset of diarrhea peaking at 150 ml/kg/day and a 15% weight loss. With total parenteral nutrition the diarrhea continued. At 12 months, his stool remained liquid, averaging 20–50 ml/kg/day. Rare bi-allelic variants in *MYO1A* were identified, including a paternally inherited 12:57037571 T/A (p.I678F; CADD score 25), and a maternally inherited 12:57044132 C/T (p.D240N; CADD score 24; Figure 3A). Myosin-1A, MYO1A, is an actin-based monomeric motor protein whose expression is limited to the intestinal tract, where it localizes almost exclusively to, and is one of the most abundant proteins in, the brush border. It is critical for normal enterocyte brush-border function^29,30^. *Myo1a* knockout mice show multiple defects in the intestinal brush border, including extensive membrane herniations and fused microvilli^31^ similar to alterations observed in the proband’s duodenal biopsy at four months of age, which showed: (i) closely packed enterocytes, some of which had a rounded teardrop shape (Figure 3B) and (ii) a reduction of MYO1A at the brush border (Figure 3C). Similarly, when overexpressed in CACO-2_BBE_ cells, the MYO1A I678F and D240N variants both exhibited aberrant localization when compared to a wild-type construct, which demonstrated strong enrichment in apical microvilli as expected (Figure 3D–F).

The proband had severe diarrhea that resolved by his 2nd birthday, consistent with the phenotype observed in *Myo1a* KO mice that survive as a result of redistribution to the brush border of other, compensatory class I myosins, MYO1C and MYO1D^31^. It would seem that the patient’s *MYO1A* variants result in the mislocalization from the microvilli of MYO1A.

### Genetic and Functional Significance of *MON1A* Variant

**Figure 4.**
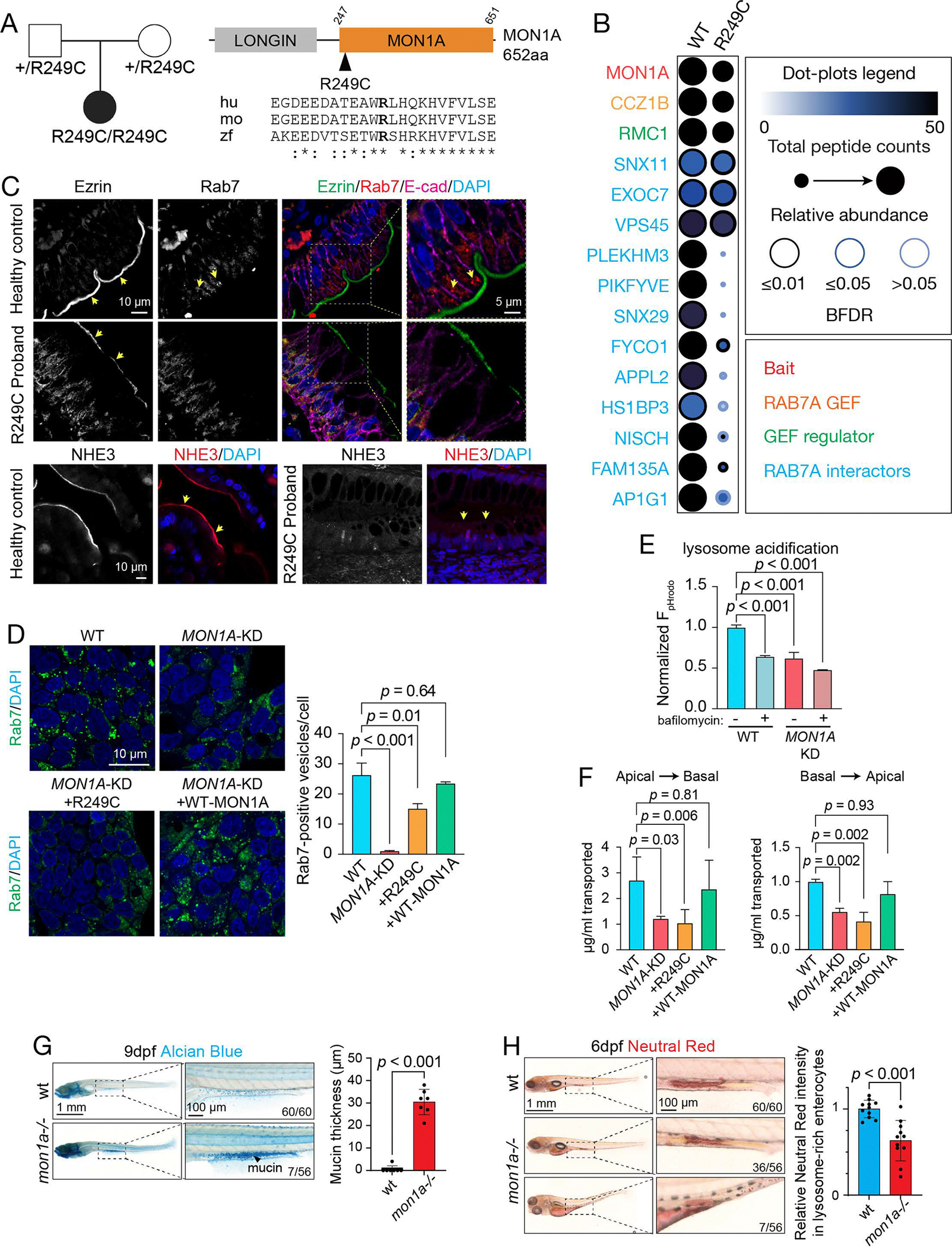
Genetic and Functional Studies of MON1A Variants. **A.** Trio WES identified biallelic variants in *MON1A* in a CODE proband. Pedigree is on the left, and a schematic illustration of the protein domain architecture is on the right, highlighting the amino acid change and its conservation across species: human (hu), mouse (mo), and zebrafish (zf). **B.** ProHits-viz Dot Plot diagram depicting BioID data for select proximity interactors of the MON1A WT and CODE variant proteins, as described in Figure 2e. **C.** (upper) Immunofluorescence images for healthy control and R249C MON1A proband tissue showing Ezrin (greyscale and green), RAB7 (greyscale and red), E-cadherin (magenta) and DAPI (blue). Arrows show reduced Ezrin at the apical brush border and loss of RAB7 positive vesicles in R249C MON1A tissue. (lower) Immunofluorescence images for healthy control and R249C MON1A proband tissue showing NHE3 (greyscale and red). Arrows show that NHE3 is localized on the apical brush border in control tissue and loss of brush border localization in R249C MON1A proband tissue. **D.** (Left) Representative images of wildtype HT-29-cells, MON1A, MON1A knockdown (MON1A-KD), or MON1A knockdown with expression of patient-variant R249C MON1A protein, or wild-type MON1A protein showing RAB7 (green) and cell nuclei (blue). (right) Summary graphs of RAB7+ vesicle size and count per cell. Statistical differences were determined by ordinary one-way ANOVA with multiple comparisons. Mean ± SEM, N = 3 experiments. **E.** Lysosome acidification in HT-29 cells using phRODO-EGF in *MON1A* wildtype or *MON1A* knockdown (*MON1A-KD*) +/− bafilomycin. Data is presented as percentage of *MON1A*. Statistical differences were determined by ordinary one-way ordinary one-way ANOVA with multiple comparisons. Mean ± SEM, N = 3 experiments. **F.** Polarized epithelial transcytosis of IgG in MDCK-FcRn cells. Summary of FcRn-dependent IgG concentrations crossing epithelium either from apical to basolateral (left) or basolateral to apical (right) in MON1A knockdown (MON1A-KD) MDCK-FcRn cells, and MON1A knockdown (MON1A-KD) MDCK-FcRn cells with expression of R249C MON1A or WT MON1A protein. Statistical differences were determined by ordinary one-way ANOVA with multiple comparisons. Mean ± SEM, N = 4 experiments. **G.** Representative bright-field images of wt and mon1a zebrafish at 9dpf. 12.5% *mon1a*−/−mutants showed high mucin secretion by alcian blue staining. Blue dots and areas indicate goblet cells and mucins in the gut. N = 56 for *mon1a*−/− at 9 dpf. N = 60 for wt at 9 dpf. The thickness of mucin was measured in the 12.5% *mon1a−/−* group, characterized by an increased mucin phenotype, and compared to wt. Statistical differences were determined by Student’s t-test. Mean ± SD, N = 7, Mean ± SD. **H.** 76.7% of *mon1a*−/− mutants displayed reduced lysosome activity in lysosome-rich enterocytes (LRE) at 6dpf. Red areas indicate the functional absorptive LREs. (N = 56 for *mon1a*−/− at 6dpf). The intensity of the red color for LRE cells was quantitated. Statistical differences were determined by Student’s t-test. N = 15, Mean ± SD.

The third CODE gene candidate, *MON1A*, was identified in a female newborn who presented with diarrhea on day three of life that developed into profound diarrhea with vomiting and failure to thrive before 1 year of age. Exome sequence analysis of this consanguineous family identified a homozygous 3:49911685 G/A *MON1A* variant (p.R249C; CADD score 27; Figure 4A). The MON1 homolog A is expressed by the cell’s secretory trafficking apparatus and is a binding partner of CCZ1^32^, together comprising a guanine nucleotide exchange factor (GEF) complex^33^ that supports RAB7A function in late endocytic trafficking^34^. As expected, BioID of MON1A identified CCZ1B, along with several proteins identified in a previous RAB7A BioID analysis^35^. While maintaining the interaction with CCZ1B, the MON1A R249C variant displayed notably reduced interactions with several RAB7A endocytic trafficking regulators (Figure 4B, Table S11). Consistent with a defect in endosome sorting caused by the disruption of MON1A/CCZ1 complex function^32^, immunohistochemistry of a duodenal biopsy from the proband demonstrated that NHE3 and EZRIN were mislocalized from the apical brush border and displayed significantly fewer RAB7-positive vesicles compared to those observed in biopsies from a healthy control (Figure 4C). Overexpression of the R249C MON1A variant resulted in reduced RAB7+ vesicle size and number, effects that could be rescued by complementation with MON1A (Figure 4D). Functional studies demonstrated that MON1A is necessary for endo-lysosome formation and acidification (Figure 4E) and similarly, polarized trafficking was altered by R249C MON1A with reduced bidirectional epithelial transcytosis of IgG in MDCK-FcRN cells (Figure 4F). Finally, *mon1a* knockout (−/−) zebrafish (Figure S3) did not show any obvious developmental defects but displayed multiple intestinal abnormalities, including morphologically atypical goblet cells with higher mucin-secretion and reduced activity of lysosome-rich enterocytes (Figure 4G–H). The R249C MON1A variant thus appears to be defective in its roles in endosomal sorting and Rab7-dependent endosome maturation in the gut, leading to a novel enteropathy.

## Discussion

We have characterized the broad genetic architecture of CODE disorders, highlighting the genes most commonly responsible and establishing the landscape of variants and heritability, including a potentially new Bedouin founder *NEUROG3* variant. The diagnostic yield of ~50% was higher than the 4% observed in monogenic IBD^11^ and other monogenic disorders^36^, and similar to some high-yield neuromuscular disorder studies^37^.

We identified causal variants in half of the CODE cases diagnosed in tertiary hospitals. It is possible that some variants in CODE genes escaped identification by exome sequence analysis. This seems particularly likely in infants with autosomal recessive disease, in whom only one variant was identified. The “missing variant” might have been detectable through genome sequencing, or obtaining better sequence coverage when exome sequencing.

It is likely that many individuals have rare or private genetic variants in novel genes. We also uncovered three novel CODE candidate genes and provided functional validation showing the variants alter protein function in relevant intestinal models that meet the criteria for reporting^38^. We used proximity-dependent biotinylation (BioID) to further support pathogenic classification of these three novel CODE genes by identifying dysregulated protein interactions in relevant intestinal pathways that are disrupted with candidate CODE variants. However, additional cases are required to further understand the pathogenesis of CODE and define genotype–phenotype correlations. Overall, we demonstrate the utility of next generation sequencing as a powerful tool to identify known and novel pathogenic variants that cause congenital diarrhea.

## Supplementary Material

supplement

supp table 8

supp table 10

supp table 11
