# Supplementary material for: The Genetic Architecture of Congenital Diarrhea and Enteropathy
Source: N Engl J Med. Author manuscript; Available in PMC 2025 Apr 3. (PMC11968080; doi:10.1056/NEJMoa2405333)
Supplement: supplement [file NIHMS2042924-supplement-supplement.docx]

**Supplementary Materials**

**The Genetic Architecture of Congenital Diarrhea and Enteropathy (CODE)**

**Table of Contents**

**Methods**

1. Genetics............................................................................................................. 2
2. BioID................................................................................................................. 4
3. Functional Studies of Candidate Genes............................................................. 6
4. Zebrafish Studies............................................................................................... 11

**Figures**

**S1.** Principal Component Analysis (PCA) of Ancestry................................................ 14

**S2.** Generation of *grwd1* Crispants Zebrafish................................................................ 15

**S3.** Generation of *mon1a* Knockout Zebrafish.............................................................. 16

**S4.** Exon Coverage of *SLC9A3* and *MYO5B*......................................... ........................ 17

**Tables**

**S1.** Demographic Characteristics of CODE Cohort............................................................. 18

**S2.** Participants and Family Structure.................................................................................. 19

**S3.** Reported and Calculated Consanguinity.................................................................. 20

**S4.** Description of WES Fields and Filters........................................................................... 21

**S5.** Quality Control Filters................................................................................................... 22

**S6.** Peddy Principal Component Analysis (PCA) – Ancestry Index................................... 23

**S7.** OMIM CODE Gene List............................................................................................... 24

**S8.** Known CODE Variant List (attached excel file)................................................... 27

**S9.** Novel CODE Genes and Variants................................................................................... 28

**S10.** GRWD1 BioID (attached excel file) ............................................................................ 29

**S11.** MON1A BioID (attached excel file) ............................................................................ 29

**References**........................................................................................................................................ 30

**Materials and Methods**

**A. Genetics**

**Human Ethics:** All human studies were conducted with ethics approval using a single NIH protocol IRB-P00027983 for University of California Los Angeles (additional IRB for UCLA 10-001653), Boston Children’s Hospital, and Vanderbilt University Medical Center in the United States, and REB 1000072542 for the Hospital for Sick Children, Canada.

**Routine Testing Statement**: All reported investigations were part of routine clinical investigations.

**Sequencing of the Congenital Diarrhea and Enteropathy Cohort:** De-identified cases were analyzed as part of the PediCODE consortium study (Pediatric Congenital Diarrhea and Enteropathies; [www.PediCoDE.org](http://www.PediCoDE.org)). All patients had a clinical diagnosis of CODE based on features outlined in^1^. Individual CODE patients or families were sequenced at their local site as previously described for each instituition^2-9^. DNA library preparation and sequencing were done at Boston Children’s Hospital through GeneDx^10^ and the Children’s Rare Disease Cohort (CRDC) initiative (PI Jay R Thiagarajah); at Vanderbilt University Medical Center (PI Sari Acra, James Goldenring); and at The Center for Applied Genomics (TCAG), the Hospital for Sick Children (Toronto; PI Yaron Avitzur). Genome and exome sequencing was done at University of California Los Angeles Sequencing Core and Psomagen (Rockville, MD, USA) at the University of California Los Angeles (PI Martín G Martín). Patients from The Hospital for Sick Children had CODE variants validated using CLIA approved sequencing. Genome Sequencing was only done at the University of California Los Angeles sequencing core.

For exome sequencing, the Burrows-Wheeler Aligner was used to alignment of raw reads to the human reference genome build  GRCh38  and variants (SNPs and short indels) were jointly called on a per sample basis using GATK Haplotype Caller (v4.1.7.0, Broad). For genomic sequencing FASTQ files were transferred via command line to Illumina BaseSpace where alignment and variant calling were done using DRAGEN.

**Quality Control:** Cross-sample contamination was evaluated using Peddy^11^. No case samples showed evidence of contamination. This was cross checked with the relevant clinical centre to exclude entry error versus potential sample swaps.  Poor-quality samples were excluded before analysis.

**Ancestry/Consanguinity Analysis:** Cases were assigned to ancestry-matched groups based on principal components inferred from the 2504 individuals in the 1000 Genomes Project (1KG) phase 3 data using peddy. Peddy was also used to estimate consanguinity.

**Variant Quality Control:** Individual variants were evaluated using a minimum genotype quality score of 20 and a read depth of at least 5. Variants with more than 90% missing genotypes in cases or families were removed. In total, 3.4 million variants passed QC.

**Sequencing Performance:** Sequence coverage per exon was calculated by extracting the read depth at each nucleotide within exonic sequences of each gene to access gene-wide sequencing performance. Box-plots for each exon of genes were generated using the ENSEMBL canonical transcript and were plotted using R.

**Variant Annotation:** Exome data was functionally annotated using the Ensembl Variant Effect Predictor (VEP version 107) according to Gencode v41 coding transcripts, using the most severe consequence on the gene. Conserved amino acids were identified using GERP. Rare variants were defined as those that were absent or had a minor allele frequency (maf) of < 1% in reference datasets (gnomADv2)^12^.

**Screening for CODE Genes:** GEMINI^13^ was used to query variants that fit with known inheritance models of OMIM^14^ genes previously associated with monogenic CODE. We first analyzed exome data for rare (gnomAD^12^ allele frequency < 0.01) and damaging (Combined Annotation Dependent Depletion (CADD) Score^15^ > 20) variants in previously published CODE genes (Table S7). For variants on the X chromosome, we also assessed possible instances of non-classical inheritance, such as non-random X-inactivation events in females.

A secondary manual filtering based on confirmatory inheritance pattern, segregation, concurrence with clinical features associated with phenotypes of known genetic disease, ClinVar annotation (<http://www.ncbi.nlm.nih.gov/clinvar/>; several variants in known genes in the CODE cohort had been previously published and/or already deposited in ClinVar), and finally pathogenicity was based on the ACMG/AMP classification^16^. An additional manual screen of the remaining cases that did not meet the above criteria was carried out to determine prior ClinVar annotated pathogenesis and to search for novel CODE genes. Reported cases were considered solved when ACMG/AMP classification pathogenic or likely pathogenic variants were identified in a CODE gene that fit the known disease phenotype and heritability similar to previously described studies^8,17^.

**B. BioID**

BioID was conducted at the Princess Margaret Cancer Centre under the supervision of Brian Raught.

**BioID**: Wildtype GRWD1 and its mutant variants were synthesized by Bio Basic (Markham, ON Canada) gene synthesis services. The synthesized cDNA ligated into the pcDNA5 FRT/TO-Flag-BirA*-MCS plasmid, was sequenced to ensure integrity. Wild-type MON1A (SPACR MGC cDNA clone) was subcloned into the pcDNA5 FRT/TO-Flag-miniTurbo-MCS plasmid, and sequenced. pcDNA5 FRT/TO-Flag-miniTurbo-MON1A-R152C mutant plasmid was generated by Q5 High-Fidelity DNA Polymerase. Plasmids were transfected into cell lines using Lipofectamine 3000 according to the manufacturer’s instructions (Thermo Fisher Scientific).

HEK293 Flp-In T-REx and Hela cell lines were cultured in DMEM supplemented with 10% heat-inactivated FBS at 37 °C and 5% CO_2_. HT-29 cells were cultured in McCoy’s 5A Medium supplemented with 10% HI-FBS and 1% antibiotic/antimycotic.

**BioID Sample Processing:** BioID was conducted as previously described^18^. In brief, cell pellets were resuspended in 10 ml lysis buffer (50 mM Tris-HCl pH 7.5, 150 mM NaCl, 1 mM EDTA, 1 mM EGTA, 1% Triton X-100, 0.1% SDS, 0.5% sodium deoxycholate, 1:500 protease inhibitor cocktail (Sigma-Aldrich), 1:1,000 benzonase nuclease (Novagen 71205-M) and incubated on an end-over-end rotator at 4°C for 1 h. The lysate was briefly sonicated to disrupt any visible aggregates, then centrifuged at 45,000×g for 30 min at 4°C. Supernatant was transferred to a fresh 15 ml conical tube. 30ul of packed, pre-equilibrated streptavidin-sepharose beads (Cytiva 17-5113-01) were added, and the mixture incubated for 3h at 4°C with end-over-end rotation. Beads were pelleted by centrifugation at 376×g for 2 min and transferred with 1 ml of lysis buffer to a fresh Eppendorf tube. Beads were washed four times with 50 mM ammonium bicarbonate (ammbic, pH 8.3) and transferred to a fresh tube for two additional washes with 1 ml of ammbic. Tryptic digestion was performed by incubating the beads with 1ug MS-grade TPCK trypsin (Promega V5280) in 200ul 50 mM ammbic (pH 8.3) overnight at 37°C. The following morning, an additional 0.5 ug trypsin was added, and the beads incubated for another 2h at 37°C. Beads were then pelleted by centrifugation at 2,000×g for 2 min, and the supernatant transferred to a fresh Eppendorf tube. Beads were washed twice with 200ul 50 mM ammbic, and the washes pooled with the eluate. The sample was lyophilized and resuspended in buffer A (0.1% formic acid). One-fifth of each sample was analysed per MS run.

**Mass Spectrometry**: High performance liquid chromatography (HPLC) was conducted using a 2 cm pre-column (Acclaim PepMap 50 mm x 100 um inner diameter) and 50 cm analytical column (Acclaim PepMap, 500 mm x 75 um diameter; C18; 2 um; 100 Å, Thermo Fisher Scientific, Waltham, MA), running a 120 min reversed-phase buffer gradient at 225 nl/min on a Proxeon EASY-nLC 1000 pump in-line with a Thermo Q-Exactive HF quadrupole-Orbitrap mass spectrometer. A parent ion scan was performed using a resolving power of 60,000, then up to the twenty most intense peaks were selected for MS/MS (minimum ion count of 1,000 for activation) using higher energy collision induced dissociation (HCD) fragmentation. Dynamic exclusion was activated such that MS/MS of the same *m/z* (within a range of 10 ppm; exclusion list size = 500) detected twice within 5 sec were excluded from analysis for 15 sec.

**Protein Identification**: Thermo .RAW files were converted to the .mzXML format using Proteowizard^19^, then searched using X!Tandem^20^ and COMET^21^ against the Human RefSeq Version 45 database (containing 36,113 entries). Data were analysed using the trans-proteomic pipeline (TPP)^22^, via the ProHits software suite (v3.3)^22^. Search parameters specified a parent ion mass tolerance of 10 ppm, and an MS/MS fragment ion tolerance of 0.4 Da, with up to 2 missed cleavages allowed for trypsin. Variable modifications of +16@M and W, +32@M and W, +42@N-terminus, and +1@N and Q were allowed. Proteins identified with an iProphet cut-off of 0.9 (corresponding to ≤1% FDR) and at least two unique peptides were analyzed with SAINT Express v.3.6.13^23^. Eighteen control runs (from cells stably expressing the FLAG-BirA* tag only) were collapsed to the four highest spectral counts for each prey, and the resulting dataset compared to experimental BioID data from two biological replicates per bait protein (each analysed with two technical replicates). High confidence interactors were defined as those with a Bayesian false discovery rate (BFDR) ≤0.01.

**C. Functional Studies of Candidate Genes**

Functional cellular studies for MYO1A were conducted at the Vanderbilt University School of Medicine under the supervision of Matthew Tyska and James R Goldenring; for MON1A at Harvard Medical School under the supervision of Jay R Thiagarajah; and GRWD1 at The Hospital for Children under the supervision of Aleixo Muise.

**Protein Modeling of Mon1a Complex:** The partial crystal structure of the yeast heterodimeric GEF Mon1a-Ccz1 in complex with Ypt7, PDB:5LDD^24^, was used as a model to visualize the human protein complex. Alpha-fold predicted protein structures of human MON1A (AF-Q86VX9), Ccz1(AF-P86791) and Rab7 (AF-P51149) were visualized in DNASTAR Lasergene Protean 3D, and were rigid body aligned using jFATCAT, a modification of the FATCAT alignment algorithm. Root mean square deviations (RMSD) for all aligned structures were assessed, and an RMSD between 0.5 – 2.0 Å corresponds to a high degree of similarity. The following RMSDs were calculated: hMON1A:sMon1 = 1.652, hCCZ1:sCcz1= 1.482, and hRAB7:sYpt7 = 1.207. The variant was highlighted in the structure with simple coloring using built in software functions.

**Cell Lines:** The MDCKII line stably expressing human β2M and HA-FcRn-EGFP (MDCK-FCRN) has been previously described^25^. Cells expressing HA-FcRN-EGFP were sorted by FACS using BD-Melody to enrich for the stably transfected FCRN receptor. All cells tested negative for mycoplasma contamination. MDCK-FCRN cells were cultured in DMEM supplemented with 10% heat-inactivated fetal bovine serum (HI-FBS) and 1% antibiotic/antimycotic (Gibco Item no. 15240062).

MON1A knockdown cell lines (MDCK-FCRN MON1A-KD or HT-29 MON1A-KD) were generated by lentiviral transduction of mission shRNA (Item no. TRCN0000420101). Lentiviral particles were generated in HEK-293T cells by using packaging plasmids psPAX2 and pVSVG. Lentivirus particles were purified and concentrated with LentiX Concentrator (Clontech 631231) and titers were determined using LentiX GloStix (Takara 631280). HT-29 cells and MDCK-FCRN cells were spin-transduced at 37°C using 1 µg/mL polybrene in OptiMEM + 10 μl of high-titer lentiparticles in PBS. Cells were washed with PBS and allowed to incubate for 3 days prior to selection with G418.

The wild-type EGFP-tagged *H.s.* MYO1A construct was generated and validated in a previous study^26^ and variants were introduced using QuikChange site-directed mutagenesis (Agilent). CACO-2_BBE_ cells were cultured at 37°C and 5% CO_2_ in DMEM with high glucose and 2 mM L-glutamine supplemented with 20% FBS. For generation of stable cell lines expressing MYO1A mutants, CACO-2_BBE_ cells were grown to 90% confluency in T25 flasks and transfected using Lipofectamine 2000 (Thermo Fisher) according to manufacturer’s instructions. Following overnight recovery, cells were reseeded in media containing 1 mg/ml G418 and grown to select for stable integration.

**Immunoprecipitation, Immunoblot Analysis and Immunofluorescence Staining:** Mounted paraffin sections from patient intestinal biopsy samples were heated to 60°C, de-paraffinized with Histo-Clear, and subsequently rehydrated through 100%, 95%, 75% ethanol, and dH_2_O washes. Sections were antigen retrieved in citrate buffer in a pressure cooker for 15 mins. The slides were washed in PBS, then incubated with Intercept protein free blocking buffer (LI-COR Item no. 927-90001).

Antibodies were incubated overnight at 4°C and washed 3 times with 1x PBS. Cells were counterstained with 4,6-diamidino-2-phenylindole (DAPI; Invitrogen, D1306) at a concentration of 2 μg/mL and mounted with Prolong Diamond Antifade. Directly conjugated primary antibodies included RAB7-594 (1:50, SCBT Item no: sc-376362), EZRIN-488 (1:100, SCBT Item no: SC32759), NHE3-565 (1:100, Novus Item no: NB110-81529R). Unconjugated MYO1A (1:100, Sigma HPA053490) antibody was used with rhodamine-conjugate anti-rabbit IgG (1:100, Jackson Immunolab).

For immunoprecipitation assays, HEK293T cells were transfected with the indicated vectors. Cells were lysed in 1 mL lysis buffer (50 mM Tris-HCl, pH 7.4, 150 mM NaCl, 1 mM EDTA, 1% Triton X-100, 10 μg/mL aprotinin, 10 μg/mL leupeptin, and 1 mM phenylmethylsulfonyl fluoride). For each immunoprecipitation, Flag affinity gel (Sigma-Aldrich, A2220) was washed with 1 mL Lysis Buffer three times, then 0.9 mL of cell lysate was incubated overnight at 4°C with constant agitation with 20 μL Flag affinity gel. Immunoprecipitated protein was washed three times with lysis buffer and resuspended in 2×SDS sample buffer. Immunoprecipitates and whole-cell lysates were analyzed by immunoblotting with rabbit polyclonal anti-Flag (Proteintech Biotechnology, 20543-1-AP) and rabbit monoclonal anti-RPL3 (Proteintech Biotechnology, 11005-1-AP). Secondary antibody was Alexa Fluor 568-conjugated goat anti-rabbit IgG (Invitrogen, A11036). Cell nuclei were stained using DAPI (Invitrogen, D1306) at a concentration of 2 μg/mL.

MDCK-FCRN and HT-29 cells that were transduced with lentivirus and selected, washed with 1x PBS and lysed with 1x RIPA buffer with protease inhibitors. Cell lysates were centrifuged at 12,000 x g for 15 mins, and supernatant collected. Samples were run on a 4-12% Bis-Tris gel, transferred to nitrocellulose membranes using the iBlot2, and immunoblotted with anti-MON1A antibody (Novus Biologicals NBP1-74123) and GAPDH-HRP (Santa Cruz Biotechnology sc-47724). Proteins were detected using SuperSignal West Femto (Fisher Scientific), and chemi-images were captured with an Azure c300 imager and quantified in ImageJ.

Cells were cultured on a 24-well Cellvis glass-bottomed dish. For immunostaining, cells were washed once with 1x PBS and fixed in 4% paraformaldehyde for 5 mins at 37°C. Cells were washed 3 times with 1x PBS, permeabilized with 1x PBS + 0.1% w/v Triton X-100 and blocked with Intercept protein-free blocking buffer (LI-Cor Item no. 927-90001).

For Hela cells, transfected cells were fixed with 4% PFA for 15 mins, then permeabilized and blocked in PBS containing Triton X-100 and 5% normal goat serum for 20 mins. Cells were then incubated with mouse monoclonal anti-Flag (Sigma-Aldrich, F3165) for 1 hr in PBS. Cells were washed three times with PBS and incubated with Alexa Fluor 488-conjugated goat anti-mouse IgG (Invitrogen, A11029) for 1 hr, washed three more times with PBS, and mounted in fluorescence mounting medium (Dako, S302380-2). A Quorum spinning disk microscope was used to perform the imaging. Briefly, the system includes an Axiovert 200 M Zeiss microscope, an ORCA-Fusion BT, Hamamatsu CCD camera (cooled), a Spectral Applied Research five-line laser module equipped with 405-, 443-, 491-, 561-, and 655-nm lines, and a MAC5000, Ludl filter wheel. The Volocity v6.3 software (Perkin Elmer) was used to control the microscope and a 63×/1.4 NA oil objective (Zeiss) was used to acquire all mages.

For CACO-2_BBE_ monolayers, cells were washed once with warm PBS and fixed with 4% paraformaldehyde in PBS for 15 min at 37°C. After fixation, cells were washed three times with PBS and permeabilized with 0.1% Triton X-100 in PBS for 15 min at room temperature (RT). Cells were washed with PBS and staining performed for 1 hour at 37°C using AlexaFluor568 phalloidin (1:200; Thermo Fisher) diluted 1:200 in PBS. Cells were washed with PBS and coverslips were mounted using ProLong Gold Antifade Mount (Thermo Fisher).

**Quantification of MYO1A variant colocalization with apical microvilli:** Confocal imaging of CACO-2_BBE_ cells was performed using a Nikon A1 laser scanning confocal microscope equipped with 488 nm, 568 nm, and 647 nm LASERs using a 60x APO TIRF oil immersion objective. Pearson correlation coefficients between EGFP and phalloidin signals were calculated using Nikon Elements; values were then plotted and statistical analyses performed using Prism (v.9.0, GraphPad). Non-parametric *t*-tests were used for statistical comparisons between MYO1A mutant and WT control data, as distributions were non-gaussian. The number of measurements for each condition from three experimental replicates are indicated in the figure legend.

**Quantification of Rab7+ Vesicles:** HT-29 cells were stained and imaged using Zeiss LSM880 +Airyscan, and images were quantified using Aivia (Aivia 8.5, Leica Microsystems, Bellevue, WA) pixel classifier with built-in cell counting recipe to assess cell count and vesicle size with modifications to previously described methodologies^27^. Briefly, images were acquired with identical pixel sizes and opened in Aivia software. The cell count recipe was trained on control images, where it performs background subtraction, intensity thresholding, segmentation, and filters objects based on size. Once the cell-count recipe was manually verified, the entire image set was processed through the recipe, and output data were spot checked and quantified. Data was plotted in Graphpad Prism 9.4, and statistics are presented as an ordinary one-way ANOVA with multiple comparison testing (Dunnett) against HT-29 wildtype cells.

**Quantification of Lysosome Acidification:** MON1A wildtype (MON1A) and MON1A knockdown (MON1A-KD) HT-29 cells were cultured on black walled 96-well plates until confluency. Lysosome acidification was carried out with modifications to previously described protocols^28^. On the day of the assay, cells were pre-treated with 200 nM Bafilomycin for 4 hrs prior to treatment with pHrodo EGF (Thermo Item no. P35374). Cells were then washed with cold HEPES pH 7.4 and incubated with 2 μg/mL of pHrodo red EGF conjugate for 60, 90 and 120 mins at 37°C. Fluorescence intensity was quantified on a Tecan Plate Reader (ex:568 nm em:585 nm). Data was plotted in Graphpad Prism 9.4, and statistics are presented as an ordinary one-way ANOVA with multiple comparison testing (Dunnett) against untreated HT-29 wildtype cells.

**FCRN-trafficking Assay:** FCRN-trafficking was assessed in MDCK-FCRN and mutant cell lines. Fc-Gaussia luciferase binding (Fc-Gluc-MN Addgene Plasmid: 198194) and non-binding (Fc-Gluc-IHH Addgene Plasmid: 198195) proteins were produced and purified as previously described^25^. Cells were plated at confluency and cultured on 0.4 μM polycarbonate transwell filters (Corning Item no. 3413) for three days until TEER reached (300 ohms x cm^2^). On the day of the assay, cells were gently washed three times with PBS and incubated with Hank’s Balanced Salt Solution (HBSS), 20 mM MES pH 6.0 with 1 ug/mL Gluc-FC (MN or IHH) on the input side, and HBSS, pH 8.0 on the output side. Transwell filters were incubated with Fc-Gluc for 90 mins at 37°C, and buffer was collected from the output side for quantification. To measure the concentration of the Fc-Gluc in the output side, a standard curve was fitted against a serial dilution of Fc-Gluc-MN and Fc-Gluc-IHH from 1 ug/mL to 1 pg/mL. Luciferase values for the standard and the experimental wells were determined using Pierce Gaussia Luciferase Glow Assay Kit (Item no. 16160) according to manufacturer’s instructions, and luciferase units were read on a Tecan Plate reader. Data is presented as ug/mL transformed to normalize between experimental replicates. Data was plotted in Graphpad Prism 9.4, and statistics are presented as an ordinary one-way ANOVA with multiple comparison testing (Dunnett) against the non-transformed control*.*

**D. Zebrafish Studies**

**Zebrafish Studies:** were carried out at the Zebrafish Genetics and Disease Models Facility, The Hospital for Sick Children, Toronto, Canada with approval from the Animal Care Committee, Animal Use Protocol #65759.

**Zebrafish Care and Transgenic Lines**: Studies were completed on larval zebrafish between 5–9 days post-fertilization (dpf). Zebrafish strains were raised and maintained using standard laboratory procedures. Embryos were obtained via natural mating and cultured in embryo E3 buffer. Tricaine (160 mg/L) was used for anesthesia. Euthanasia of zebrafish at different stages is carried out by the following methods in accordance with the AVMA guidelines on euthanasia. For zebrafish >8 dpf, tricaine overdose (500mg/L) and ice bath (0– 4º C) was used for euthanasia for 10 mins following cessation of opercular (i.e., gill) movement. For zebrafish 0–7dpf, ice bath followed by immersion in a dilute sodium hypochlorite for 5 mins was used for euthanasia.

CRISPR/Cas9 system was utilized to knockout *mon1a.* The targeting sequence 5’- AGATCAGAGAGTCCGACCCC- 3’ was designed by CHOPCHOP. mRNA (150 pg) encoding a zebrafish-codon-optimized Cas9 together with gRNA (100 pg) were injected at the one-cell stage. Microinjections and F0, F1 screen were carried out according to standard protocols. The mutant allele with 7bp insertion and 3bp deletion were identified by High-Resolution Melting (HRM) analysis and confirmed by Sanger sequencing. The 7bp insertion and 3bp deletion results in a premature stop codon on exon1. For genotyping, DNA fragment was amplified with primers (F1:5′- CGGCATATGTCCACAGCAAG -3′ and R1: 5′- CTAACCTGGCTCAGTGCCC -3′) and further confirmed by Sanger sequencing.

The CRISPR/Cas9 system was utilized to knockout *grwd1*. The targeting sequences are: grwd1 gRNA1: 5’- GATACATGCGGTAAGCGGAG-3’ and grwd1 gRNA2: 5’- ACTCCACCGACAGATCCCAT-3’ were designed by CHOPCHOP. 100 pg of each gRNA and 150 pg of Cas9 mRNA were injected into embryos at the one-cell stage. The F0 larvae were transferred to the nursery (n=96) at 5dpf. All died at 5dpf-15dpf.  Therefore, we used first-generation (F0) mosaic *grwd1* mutant zebrafish by CRISPR (crispants^29^).

**Alcian Blue Staining:** Alcian blue staining（Sigma [A3157](https://www.sigmaaldrich.com/CA/en/product/sigma/a3157)) was used for optimal visualization of goblet cells and mucin secretion in the gut of the embryos. Embryos were collected and raised in a petri dish with E3 medium supplemented with 1-phenyl 2-thiourea (PTU) to improve optical transparency. After 5-8dpf, zebrafish larvae were treated with tricaine and euthanized by rapid cooling of the plates on ice. Larvae were fixed in 4% paraformaldehyde (PFA) at 4°C temperature for 48 hrs. Fixed larvae were washed three times with PBS for 10 mins, then rinsed with acid alcohol (70% ethanol, 1% HCl) for 5 mins. Then, larvae were stained with 0.1% alcian blue in 70% ethanol, 1% HCl at room temperature for 3.5 hrs. Alcian blue medium was removed, and the larvae were washed in 70% ethanol, 1% HCl at room temperature overnight. The next day, larvae were washed with PBS three times for 10 mins at 21°C. Stained larvae were stored in PBS or 70% glycerol at 4°C. When imaging, stained embryos were positioned in 70% glycerol and the trunk with gut photographed in a lateral view using Zeiss Axio zoom imager. Images were analyzed with Fiji. The thickness of mucin and the size of goblet cells were measured and quantified. *t*-tests were used to compare the means of two groups.

**Neutral Red Staining:** Neutral Red staining (Sigma [72210](https://www.sigmaaldrich.com/CA/en/product/sial/72210)) was used for optimal visualization of Lysosome-rich enterocytes (LRE) in the gut of living zebrafish larvae. Embryos were collected and raised in a petri dish with E3 medium with PTU. At 8dpf, zebrafish were placed in 10 mm petri dish. Neutral Red solution was added to the medium to reach a final concentration of 3 ug/mL. Dishes were incubated at 28.5°C for 4 hrs, then moved into 50 mL fresh egg water and anesthetized with tricaine, followed by immediate imaging. Immobilized larvae were positioned in 3% methylcellulose and guts were imaged in a lateral view using a Zeiss Axio zoom imager. The intensity of the neutral red stained LRE cells was measured by Fiji. The color of the image was inverted, the gut lumen was traced, and the mean intensity was measured using Fiji software. The mean intensity of images from the anal end of the gut was subtracted to remove background signal. Relative intensity was calculated by normalizing to the wt group. *t*-tests were used to compare the means of two groups.

**Zebrafish Length Measurement:** Zebrafish larvae at 8dpf were anesthetized using tricaine solution (160 µg/mL). Once anesthetized, zebrafish were positioned in a medium of 3% methylcellulose to facilitate imaging with an Axio Zoom microscope. Body and gut lengths were measured by Fiji and analyzed by GraphPad. *t*-tests were used to compare the means of two groups.

**Histology H&E Staining:** Zebrafish larvae were fixed in 4% (v/v) PFA/PBS for 48 hrs at 4℃, followed by alcian blue staining as described above. The samples were then dehydrated in gradient ethanol and embedded in paraffin. 3 μm-lengthwise sections were prepared and stained with H&E according to standard protocols by UHN Pathology Research Program. The measurement of goblet cell area was analyzed by Fiji using the selection tool Region of Interest (ROI) to trace the boundary around individual goblet cells (n=30), and the average area was compared between two groups. Two-tailed *t*- test were used for statistical analysis.

**Electron Microscope Analysis:** Zebrafish larvae at 8 dpf were anesthetized by rapid cooling on ice water for 10 mins, then fixed in Karnovsky’s fixative overnight at 4°C. Fixed larvae were rinsed in 0.1M sodium cacodylate buffer, post-fixed in 1% osmium tetroxide in buffer for 2 hrs, dehydrated in an ethanol series, and embedded in Quetol-Spurr resin. 70 nm sections were cut with a Leica UC7 ultramicrotome, stained with uranyl acetate and lead citrate for 20 mins, washed and dried, and viewed with either an FEI Tecnai 20 transmission electron microscope (Technai, Oregon, USA) or a JEOL JEM 1200EX TEM (JEOL, Massachusetts, USA). Images were obtained using Gatan Digital Micrograph acquisition software for Orius CCD Camera or AmtV542.

**RNAseq:** RNA was extracted from pooled wildtype or *grwd1* crispant zebrafish larvae at 8 dpf (n=15) using the RNeasy Mini Kit (QIAGEN) as described previously^30^. Raw sequence FASTQ files were assessed for quality control using FastQC v0.12.1 and MultiQC v1.15^31^, trimmed to remove adapters using Trimmomatic v0.39^32^, and aligned to the zebrafish reference genome GRCz11 using HISAT2 v2.2.1^33^. Aligned SAMs were converted to BAMs, sorted and indexed using samtools v1.20^34^. Reads in genes were counted using featureCounts v2.0.6^35^. Differential expression analysis was carried out in R v4.4.0 using the DESeq2 v1.44.0 package^36^ with genotype (*grwd1* vs wt) as the single design variable. Genes with an adjusted Wald test *p*-value of 0.05 or less were considered differentially expressed. Finally, output results from DESeq2 were annotated with gene metadata from Ensembl using the biomaRt package^37^. Graphs were generated with JMP 16 (SAS Institute).


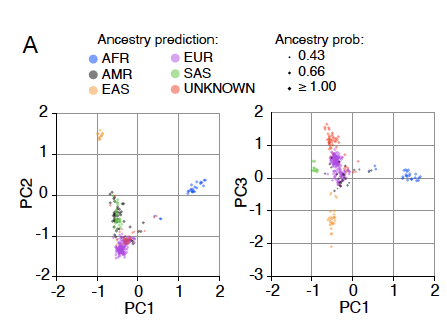


**Figure S1.** **Principal Component Analysis (PCA) of Ancestry**.

PCA was conducted on each patient, and ancestry predictions were made based on SVM (support vector machine) model trained on the 1000 genome samples (1kg) into five superpopulations: Africans (AFR), Americans (AMR), East Asians (EAS), Europeans (EUR) and South Asians (SAS).

**
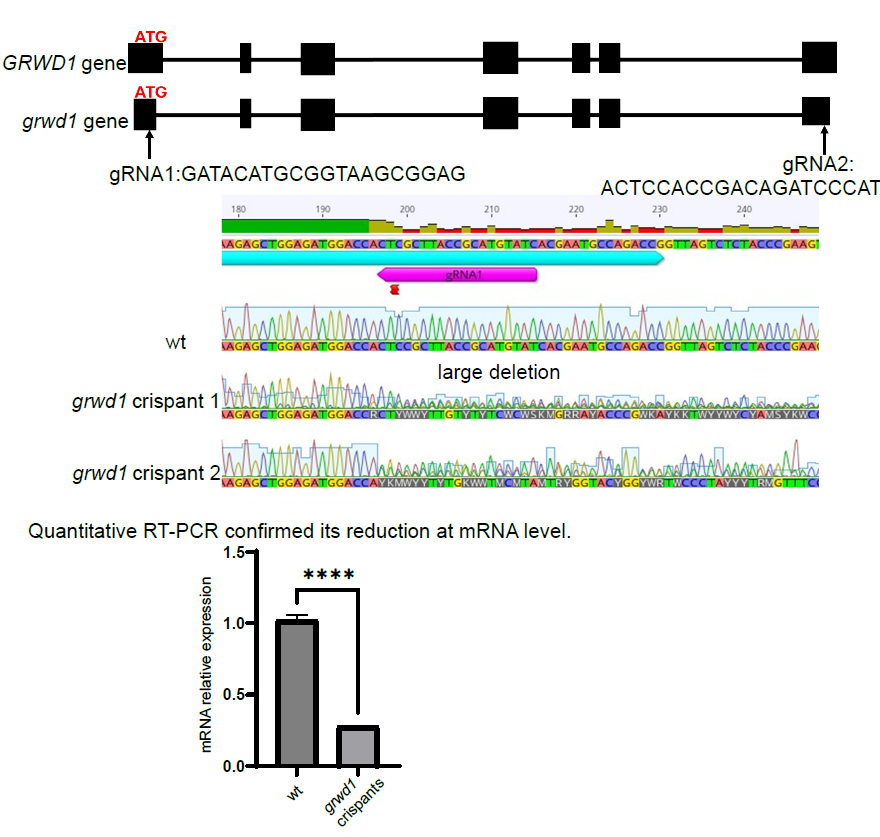
**

**Figure S2. Generation of *grwd1* Crispants Zebrafish**

Top: Human *GRWD1* and its zebrafish homolog, *grwd1*, consist of seven exons with the translation start site located in exon 1.

Middle: *grwd1* crispants generation: CRISPR/Cas9 genome editing was employed to generate a *grwd1* knockout mutant. *grwd1* gRNA1 targeted exon 1 and grwd1 gRNA2 targeted exon 7, resulting in a large deletion between them. The first-generation (F0) larvae died within 5dpf-15dpf.  Therefore, we used F0 mosaic *grwd1* mutant zebrafish (crispants) before 15dpf for experiments.

Bottom: Molecular validation: Sanger sequencing confirmed the mutations and real-time PCR demonstrated downregulation of grwd1mRNA, suggesting nonsense-mediated RNA decay.

**Figure S3. Generation of *mon1a* Knockout Zebrafish**

Top: Human *MON1A* and its zebrafish homolog, *mon1a*, consist of six exons with the translation start site located in exon 2.

Middle: *mon1a*-/- generation: CRISPR/Cas9 genome editing was employed to generate an *mon1a* knockout. The guide RNA targeted exon 2 resulting in a 7 bp insertion and 3bp deletion, generating an early stop codon on exon2.

Bottom: Molecular validation: Sanger sequencing and high-resolution melting (HRM) analysis confirmed the mutations.

**Figure S4. Exon Coverage of *SLC9A* and *MYO5B.***

Box and whisker plot - the line in the graph represents median read depth. The error bar extends the box by 1.5 * inter-quartile range. (red x) indicates coverage of proband 5 with missed *SLC9A3* variant.

| **Demographic Characteristics** | **Total CODE Cases (n = 139)** |
| --- | --- |
| **CODE Sex and Gender Overview** | Autosomal Recessive inherited CODE disorders affect both males and females equally. X-linked disorders affect males^38-42^. |
| **Study Sex** (n, %) |  |
| Male | 63 (45.3%) |
| Female | 76 (54.7%) |
| **Age Overview*** | CODE disorders are generally diagnosed before two years of age with most patients diagnosed within a few weeks of birth^38-42^. Some enteropathy disorders may be diagnosed later in childhood^38-42^. |
| **CODE Geography and Ethnic Groups Overview** | CODE disorders are found in people from all over the world. Several ethnic groups have founder variants including a *MYO5B* pathogenic variant in Navajo Indigenous Americans^43^, an *EPCAM* pathogenic variant found in the Arabic Gulf^44^, a *DGAT1* pathogenic variant in Ashkenazi Jewish families^45^, and a *NEUROG3* pathogenic variant in Bedouin families described here. |
| **Study Ancestry**^ (n, %) |  |
| Europe (EUR) | 81 (58.3%) |
| East Asia (EAS) | 3 (2.2%) |
| Africa (AFR) | 10 (7.2%) |
| South Asia (SAS) | 9 (6.5%) |
| American (AMR) | 23 (16.5%) |
| Unknown | 13 (9.4%) |

**Table S1.** **Demographic Characteristics of CODE Cohort**

*All patients were reported to be diagnosed at < 1 year of age. ^ Cases were assigned to ancestry-matched groups based on principal components inferred from the 2504 individuals in the 1000 Genomes Project (1KG) phase 3 data using peddy.

| **Participants** | **Number** |
| --- | --- |
| Index cases | 129 |
| Affected siblings | 10 |
| Unaffected siblings | 13 |
| Mothers | 87 |
| Fathers | 75 |
| Other family members | 7 |

| **FAMILY STRUCTURE** | **TOTAL COHORT** | |
| --- | --- | --- |
|  | N (n=139) | % |
| Singleton | 31 | 22.3% |
| Duo | 36 | 25.9% |
| Trio | 55 | 39.6% |
| Quad | 9 | 6.5% |
| Quint | 7 | 5.0% |
| > Quint | 1 | 0.7% |

**Table S2. Participants and Family Structure.**

| **Relationship Coefficient** | **Clinical Report** | **Affected Sibling** |
| --- | --- | --- |
| 0.1567 | Unknown | No |
| 0.1814 | Unknown | No |
| 0.4928 | Yes | No |
| 0.09645 | Yes | No |
| 0.1775 | Yes | Yes |
| 0.1059 | Unknown | No |
| 0.1567 | Yes | No |
| 0.1418 | No | No |
| 0.2389 | No | No |
| 0.1248 | Unknown | No |
| 0.2823 | Yes | No |
| 0.1167 | No | No |
| 0.1217 | Unknown | No |
| 0.1424 | Unknown | No |

**Table S3. Reported and Calculated Consanguinity.**

Consanguinity relationship between reported and computationally calculated evidence of consanguinity as derived from peddy. A relationship coefficient >0.1 was suggestive of consanguinity.

| **Fields** |
| --- |
| Consequence |
| Codons |
| Amino_acids |
| Gene |
| SYMBOL |
| Feature |
| EXON |
| PolyPhen |
| SIFT |
| Protein_position |
| BIOTYPE |
| CANONICAL |
| CCDS |
| RadialSVM_score |
| RadialSVM_pred |
| LR_score |
| LR_pred |
| CADD_raw |
| CADD_phred |
| Reliability_index |
| LoF |
| LoF_filter |
| LoF_flags |

**Table S4a**. Outline of WES fields utilised in pipeline.

| **Inheritance Mode** |
| --- |
| Autosomal recessive (AR) |
| De_novo (DN) |
| Autosomal dominant (AD) |
| X-linked recessive (XL) |
| Compound hets (CH, ARa/ARb) |

**Table S4b.** Explanation of WES inheritance models.

| **Filter** |
| --- |
| impact != synonymous_variant |
| impact severity != LOW |
| max_aaf_all < 0.01 |

**Table S4c.** Description of WES filters employed in pipeline

| 1. **Sex check**   Sex check performs a comparison between the sex reported in the ped file and that inferred from the genotypes on the  non-PAR regions of the X chromosome.  • sample id: sample from ped.  • error: boolean indicating whether there is a mismatch between X genotypes and ped sex.  • het count: number of heterozygote calls  • hom alt_count: number of homozygous-alternate calls  • hom ref_count: number of homozygous-reference calls  • het ratio: ratio of het_ ount / hom alt_count. Low for males, high for females  • ped sex: sex from .ped file  • predicted_ sex: sex predicted from rate of hets on chrX. |
| --- |
| 1. **Het_check**   Het check does general QC including rate of het calls, allele-balance at het calls, mean and median depth, and a PCA  projection onto thousand genomes.  • sample_id: sample from ped.  • sampled_sites: number of sites sampled (sufficient call-rate across samples and depth in this sample)  • mean/median_depth: mean/median depths for the sites tested.  • depth_outlier: boolean indicating that this sample’s depth is considered an outlier relative to the other samples.  • het_count: number of heterozygote calls in sampled sites.  • het_ratio: proportion of sites that were heterozygous.  • ratio_outlier: boolean indicating that the het_ratio was outside what is normally seen.  • idr_baf: inter-decile range (90th percentile - 10th percentile) of b-allele frequency. Distribution of all sites of alts / (ref + alts) and then report the difference between the 90th and the 10th percentile. Large values indicated likely sample contamination.  • p10/p90: the numbers used to calculate idr_baf. |
| 1. **Ped_check**   Ped check compares the relatedness of 2 samples as reported in a .ped file to the relatedness inferred from the genotypes and ~25K sites in the genome.  • sample_a/sample_b: the samples indicating the pair in question.  • n: the number of sites that was used to predict the relatedness.  • rel: the relatedness calculated from the genotypes.  • pedigree_relatedness: the relatedness reported in the ped file.  • rel_difference: difference between the preceding 2 columns.  • ibs0: the number of sites at which the 2 samples shared no alleles (should approach 0 for parent-child pairs).  • ibs2: the number of sites and which the 2 samples where both hom-ref, both het, or both hom-alt.  • shared_hets: the number of sites at which both samples were hets.  • hets_a/b: the number of sites at which sample_a/b was het.  • pedigree_parents: boolean indicating that this pair is a parent-child pair according to the ped file.  • predicted_parents: boolean indicating that this pair is expected to be a parent-child pair according to the ibs0 (<0.012) calculated from the genotypes.  • parent_error: boolean indicating that the preceding 2 columns don’t match  • sample_duplication_error: boolean indicating that rel > 0.75 and ibs0 < 0.012 |
| 1. **PCA: Ancestry**   • PC1/PC2/PC3/PC4: the first 4 values after this sample was projected onto the thousand genomes principle components.  • ancestry-prediction: one of AFR AMR EAS EUR SAS UNKNOWN where it is unknown if ancestry-prob < 0.65  for the highest population  • ancestry-prob: the highest probability from the SVM for any ancestry (between 0 and 1). |

**Table S5.** **Quality Control Filters**.

| **African ancestry** |
| --- |
| Esan in Nigeria Esan, ESN |
| Gambian in Western Division, Mandinka Gambian GWD |
| Luhya in Webuye, Kenya Luhya LWK |
| Mende in Sierra Leone Mende MSL |
| Yoruba in Ibadan, Nigeria Yoruba YRI |
| African Caribbean in Barbados Barbadian ACB |
| People with African Ancestry in Southwest USA African American SW ASW |
| **Americas** |
| Colombians in Medellin, Colombia Colombian CLM |
| People with Mexican Ancestry in Los Angeles, CA, USA Mexican American MXL |
| Peruvians in Lima, Peru Peruvian PEL |
| Puerto Ricans in Puerto Rico Puerto Rican PUR |
| **East Asian ancestry** |
| Chinese Dai in Xishuangbanna, China Dai Chinese CDX |
| Han Chinese in Beijing, China Han Chinese CHB |
| Southern Han Chinese Southern Han Chinese CHS |
| Japanese in Tokyo, Japan Japanese JPT EAS |
| Kinh in Ho Chi Minh City, Vietnam Kinh Vietnamese KHV |
| **European ancestry** |
| Utah residents (CEPH) with Northern and Western European ancestry CEPH CEU |
| British in England and Scotland British GBR |
| Finnish in Finland Finnish FIN |
| Iberian Populations in Spain Spanish IBS |
| Toscani in Italia Tuscan TSI |
| **South Asian ancestry** |
| Bengali in Bangladesh Bengali BEB |
| Gujarati Indians in Houston, TX, USA Gujarati GIH |
| Indian Telugu in the UK Telugu ITU |
| Punjabi in Lahore, Pakistan Punjabi PJL |
| Sri Lankan Tamil in the UK, Tamil, STU |

**Table S6. Peddy Principal Component Analysis (PCA) – Ancestry Index**.

Peddy ancestry superpopulation groups based on the known ancestry of 2,504 individuals collected from diverse world populations as part of the 1000 Genomes Project and used to predict the ancestry of each individual in the study cohort.

| **GENE** | **GENE NAME** | **DISEASE ASSOCIATIONS** | **OMIM** | **CHROMOSOME** | **INHERITANCE** |
| --- | --- | --- | --- | --- | --- |
| ***ADAM17*** | A Disintegrin and Metalloproteinase Domain 17 | Inflammatory skin and bowel disease | 603639 | 2p25.1 | AR |
| ***AGR2*** | Anterior Gradient 2 | EAGLES syndrome | 606358 | 7p21.1 | AR |
| ***AIRE*** | Autoimmune Regulator | Respiratory infections, recurrent, and failure to thrive with or without diarrhea | 607358 | 21q22.3 | AR |
| ***ALG6*** | Alpha‐1,3‐Glucosyltrasferase | Congenital Disorder of Glycosylation | 604566 | 1p31.3 | AR |
| ***ALG8*** | Alpha‐1,3‐Glucosyltrasferase | Congenital Disorder of Glycosylation type 1h | 608104 | 11q14.1 | AR |
| ***ANGPTL3*** | Angiopoietin‐like 3 | Hypobetalipoproteinemia | 605019 | 1p31.3 | AR |
| ***AP1S1*** | Adaptor‐Related Protein Complex 1, Sigma‐1 Subunit | MEDNIK syndrome | 609313 | 7q22.1 | AR |
| ***APOB*** | Apolipoprotein B | Hypobetalipoproteinemia | 615558 | 2p24.1 | AR |
| ***ARX*** | Aristaless‐Related Homeobox | X‐linked lissencephaly and MR | 300215 | Xp21.3 | XR |
| ***CARD11*** | Caspase Recruitment Domain‐Containing Protein 11 | Immunodeficiency | 607210 | 7p22.2 | AD |
| ***CARMIL2*** | Capping Protein Regulator and Myosin 1 Linker 2 | Immunodeficiency | 610859 | 16q22.1 | AR |
| ***CD55*** | Decay Accelerating Factor for Complement (DAF) | Complement Hyperactivation, Angiopathic Thrombosis and Protein Losing Enteropathy (CHAPLE) | 226300 | 1q32.2 | AR |
| ***CYBB*** | Cytochrome b(‐245) Beta Subunit | Chronic Granulomatous Disease | 300481 | Xp21.1‐p11.4 | XL |
| ***DEF6*** | Guanine Nucleotide Exchange Factor | Immunodeficiency 87 and autoimmunity | 610094 | 6p21.31 | AR |
| ***DGAT1*** | Diacyglycerol O‐Acyltransferase 1 | DGAT1 Deficiency | 615863 | 8q24.3 | AR |
| ***DKC1*** | Dyskerin | Hoyeraal‐Hreidarsson Syndrome | [305000](https://can01.safelinks.protection.outlook.com/?url=https%3A%2F%2Fwww.omim.org%2Fentry%2F305000&data=05%7C02%7Caleixo.muise%40sickkids.ca%7Cbc284218b99244fa15a908dca6737263%7C3961553ff47e49eb9f6ccf8518914e9a%7C0%7C0%7C638568261974364067%7CUnknown%7CTWFpbGZsb3d8eyJWIjoiMC4wLjAwMDAiLCJQIjoiV2luMzIiLCJBTiI6Ik1haWwiLCJXVCI6Mn0%3D%7C0%7C%7C%7C&sdata=KSIU7Nj%2FU3CQSAn0oYVxe3yay46oxLhUYwqWIiny4NY%3D&reserved=0) | Xq28 | XR |
| ***EGFR*** | Epidermal Growth Factor Receptor | EGFR Deficiency | 616069 | 7p11.2 | AR |
| ***ELANE*** | Elastase Neutrophil‐Expressed | Neutropenia | 130130 | 19p13.3 | AD |
| ***EPCAM*** | Epithelial Cellular Adhesion Molecule | Tufting Enteropathy | 613217 | 2p21 | AR |
| ***FOXP3*** | Forkhead Box P3 | Imunodysregulation, Polyendocrinopathy and Enteropathy, X‐linked | 300292 | Xp11.23 | XL |
| ***GUCY2C*** | Guanylate Cyclase 2C | Congenital sodium diarrhea | 614616 | 12p12.3 | AD |
| ***IKBKG*** | Inhibitor of Nuclear Factor Kappa‐B Kinase | NEMO | 300248 | Xq28 | XL |
| ***IL10RA*** | Interleukin 10 Receptor Alpha | Inflammatory bowel disease | 146933 | 11q23.3 | AR |
| ***IL10RB*** | Interleukin 10 Receptor Beta | Inflammatory bowel disease | 612567 | 21q22.11 | AR |
| ***IL2RA*** | Interleukin 2 Receptor Alpha | Immunodeficiency with lymphoproliferation and autoimmunity | 606367 | 10p15.1 | AR |
| ***KMT2D*** | Lysine‐Specific Methyltransferase 2D | Kabuki Syndrome | 602113 | 12q13.12 | AD |
| ***LCT*** | Lactase | Congenital Lactase Deficiency | 223000 | 2q21.3 | AR |
| ***LIPA*** | Lipase A Lysosomal Acid | Wolman's Disease | 620151 | 10q23.31 | AR |
| ***LRBA*** | Lipopolysaccharide‐Responsive Beige‐Like Anchor Protein | Immunodeficiency | 606453 | 4q31.3 | AR |
| ***MALT1*** | Mucosa‐Associated Lymphoid Tissue Lymphoma Translocation Gene 1 | Immunodeficiency | 604860 | 18q21.32 | AR |
| ***MPI*** | Mannosephosphate Isomerase | Congenital Disorder of Glycosylation type 1b | 602579 | 15q24.1‐q24.2 | AR |
| ***MTTP*** | Microsomal Triglyceride Transfer Protein | Abetalipoproteinemia | 157147 | 4q23 | AR |
| ***MVK*** | Mevalonate Kinase | Hyper‐IgD Syndrome | 260920 | 12q24.11 | AR |
| ***MYO5B*** | Myosin VB | Microvillus Inclusion Disease (MVID) | 251850 | 18q21.1 | AR |
| ***NCF2*** | Neutrophil Cytosolic Factor 2 | Chronic Granulomatous Disease 2 | 233710 | 1q25.3 | AR |
| ***NEUROG3*** | Neurogenin 3 | Enteric Anendocrinosis | 610370 | 10q22.1 | AR |
| ***NFKBIA*** | Nuclear Factor Kappa‐B Inhibitor Alpha | Ectodermal Dysplasia and Immunodeficiency | 612132 | 14q13.2 | AD |
| ***PARN*** | Polyadenylate‐Specific Ribonuclease | Dyskeratosis Congenita | 604212 | 16p13.12 | AR |
| ***PCSK1*** | Proprotein Convertase Subtilisin Kexin‐Type 1 | Enteric Dysendocrinosis | 600955 | 5q15 | AR |
| ***PERCC1*** | Proline‐and Glutamate‐Rich Protein with Coiled‐Coil Domain 1 | Intractable Diarrhea of Infancy syndrome | 618662 | 16p13.3 | AR |
| ***PLVAP*** | Plasmalemma Vesicle‐Associated Protein | Protein‐Losing Enteropathy | 618183 | 19p13.11 | AR |
| ***RFX6*** | Regulatory Factor X | Mitchell‐Riley Syndrome | 615710 | 6q22.1 | AR |
| ***RIPK1*** | Receptor‐Interacting Serine/ Threonine Kinase 1 | Immunodeficiency with autoinflammation | 603453 | 6p25.2 | AR |
| ***RNF31*** | Ring Finger Protein 31 | Immunodeficiency with autoinflammation | 612487 | 14q12 | AR |
| ***RTEL1*** | Regulator of Telomere Elongation Helicase 1 | Dyskeratosis Congenita | 608833 | 20q13.33 | AR |
| ***SAR1B*** | Secretion‐Associated Ras‐Related GTPase 1B | Chylomicron retention Disease | 246700 | 5q31.1 | AR |
| ***SBDS*** | Shwachman‐Diamond syndrome | Shwachman‐ Diamond Syndrome | 260400 | 7q11.21 | AR |
| ***SI*** | Sucrase‐Isomaltase | Sucrase‐Isomaltase Deficiency | 222900 | 3q26.1 | AR |
| ***SKIV2L*** | SK12 Subunit of Superkiller Complex | Trichohepatoenteric Syndrome 2 (THES2) | 614602 | 6p21.33 | AR |
| ***SLC10A2*** | Solute Carrier Family 10 Member 2 | Primary Bile Acid Diarrhea | 613291 | 13q33.1 | AR |
| ***SLC26A3*** | Solute Carrier Family 26 Member 3 | Congenital Chloride Diarrhea | 214700 | 7q22.3‐q31.1 | AR |
| ***SLC39A4*** | Solute Carrier Family 39 Member 4 | Acrodermatitis Enteropathy | 201100 | 8q24.3 | AR |
| ***SLC51B*** | Solute Carrier Family 51 Subunit Beta | Primary Bile Acid Diarrhea | 612085 | 15q22.31 | AR |
| ***SLC5A1*** | Solute Carrier Family 5 Member 1 | Glucose‐Galactose Malabsorption | 606824 | 22q12.3 | AR |
| ***SLC7A7*** | Soulte Carrier Family 7 | Lysinuric Protein Intolerance | 222700 | 14q11.2 | AR |
| ***SLC9A3*** | Solute Carrier Family 9 Member 3 | Congenital Sodium Diarrhea | 616868 | 5p15.33 | AR |
| ***SLCO2A1*** | Solute Carrier Organic Anion Transporter Family Member 2A1 | Enteropathy | 614441 | 3q22.1‐q22.2 | AR |
| ***SPINT2*** | Serine Peptidase Inhibitor Kunitz‐Type 2 | Syndromic Na+ diarrhea /Tufting Enteropathy | 270420 | 19q13.2 | AR |
| ***STX3*** | Syntaxin 3 | Microvillus Inclusion Disease | 619445 | 11q12.1 | AR |
| ***STXBP2*** | Syntaxin-Binding Protein 2 | Familial Hemophagocytic Lymphohistiocytosis 5 | 613101 | 19p13.2 | AR |
| ***TERT*** | Telomerase Reverse Transcriptase | Dyskeratosis Congenita, Hoyeraal‐Hreidarsson Syndrome | 613989 | 5p15.33 | AR |
| ***TINF2*** | Terf1‐Interacting Nuclear Factor 2 | Dyskeratosis Congenita | 604319 | 14q12 | AD |
| ***TMPRSS15*** | Transmembrane Protease Serine 15 | Enterokinase deficiency | 226200 | 21q21.1 | AR |
| ***TNFAIP3*** | Tumor Necrosis Factor‐Alpha ‐Induced Protein 3 | Autoinflammatory syndrome | 616744 | 6q23.3 | AD |
| ***TREH*** | Trehalase | Trehalase deficiency | 612119 | 11q23.3 | AR |
| ***TTC37*** | Tetratricopeptide repeat domain 37 | Trichohepatoenteric Syndrome 1 (THES1) | 222470 | 5q15 | AR |
| ***TTC7A*** | Tetratricopeptide Repeat Domain‐Containing Protein 7A | TTC7A deficiency | 243150 | 2p21 | AR |
| ***UBR1*** | UBR1 Ribosome Biogenesis 1 | Johanson-Blizzard syndrome | [243800](https://www.omim.org/entry/243800) | 21q22.11 | AR |
| ***UNC45A*** | UNC45 Myosin Chaperone | Microvillus Inclusion Disease | 619377 | 15q26.1 | AR |
| ***WAS*** | Wiskott‐Aldrich Syndrome | Wiskott‐Aldrich Syndrome | 301000 | Xp11.23 | XL |
| ***WNT2B*** | Wingless‐Type MMTV Integration Site Family Member 2B | WNT2B Deficiency | 618168 | 1p13.2 | AR |
| ***XIAP*** | X ‐linked Inhibitor of Apoptosis | X‐linked Lymphoproliferative Syndrome | 300635 | Xq25 | XL |

**Table S7. OMIM CODE Gene List**

**S8.** Known CODE Variant List (attached excel file)

| **# of affected family members** | 2 | 2 | 1 | 1 | 1 |
| --- | --- | --- | --- | --- | --- |
| **inheritance** | Mother | Father | Mother / Father | Mother | Father |
| **proband zygosity** | heterozygous | heterozygous | homozygous | heterozygous | heterozygous |
| **Candidate gene** | **GRWD1** | **GRWD1** | **MON1A** | **MYO1A** | **MYO1A** |
| **mRNA change** | NM_031485.3:c.920A>G | NM_031485.3:c.1102G>T | NM_032355.4:c.745C>T | NM_005379.4:c.718G>A | NM_005379.4:c.2032A>T |
| **protein change** | p.H307R | p.V368F | p.R249C | p.D240N | p.I678F |
| **CADD score v1.6** | 24.9 | 28.0 | 27.0 | 23.7 | 24.6 |
| **GRCh38 chr:pos ref/alt** | 19:48451128 A/G | 19:48452786 G/T | 3:49911685 G/A | 12:57044132 C/T | 12:57037571 T/A |
| **gnomAD maf v4 (# of homozygotes)** | 0.00024 (0) | 0.0000021 (0) | 0.000033 (0) | 0.000021 (0) | 0.0031 (21) |
| **protein domain** | WD40 repeat (blade 5) | WD40 repeat (blade 6) | Longin domain 1 | Myosin Motor Head | Myosin Motor Head |
| **exon** | 6 | 7 | 3 | 9 | 19 |
| **dbSNP ID** | rs201243351 | rs578057612 | rs555274206 | rs775862921 | rs151269703 |
| **# of family members sequenced** | 4 | 4 | 3 | 3 | 3 |
| **ACMG classification** | Likely Pathogenic | Likely Pathogenic | Likely Pathogenic | Likely Pathogenic | VUS |
| **ACMG classification evidence codes** | PS3  PM1  PM2  PM3  PP3  — | PS3  PM1  PM2  PM3  PP3  — | PS3  PM1  PM2  PM3  PP3  — | PS3  PM1  PM2  PM3  PP3  — | PS3  —  —  PM3  PP3  BS2 |
| **HPO term ‐ chronic diarrhea** | 0002028 | 0002028 | 0002028 | 0002028 | 0002028 |
| **HPO term ‐ vomiting** | — | — | 0002013 | — | — |
| **HPO term ‐ failure to thrive in infancy** | — | — | 0001531 | — | — |

**Table S9. Novel CODE Genes and Variants**

**Table S10. GRWD1 BioID** (attached excel file)

**Table S11.** **MON1A BioID** (attached excel file)

**References**

1. Thiagarajah JR, Kamin DS, Acra S, et al. Advances in Evaluation of Chronic Diarrhea in Infants. Gastroenterology 2018;154(8):2045-2059 e6. DOI: 10.1053/j.gastro.2018.03.067.

2. Wang L, Aschenbrenner D, Zeng Z, et al. Gain-of-function variants in SYK cause immune dysregulation and systemic inflammation in humans and mice. Nat Genet 2021;53(4):500-510. (In eng). DOI: 10.1038/s41588-021-00803-4.

3. Zhang YJ, Jimenez L, Azova S, et al. Novel variants in the stem cell niche factor WNT2B define the disease phenotype as a congenital enteropathy with ocular dysgenesis. Eur J Hum Genet 2021;29(6):998-1007. (In eng). DOI: 10.1038/s41431-021-00812-1.

4. Pathak SJ, Mueller JL, Okamoto K, et al. EPCAM mutation update: Variants associated with congenital tufting enteropathy and Lynch syndrome. Hum Mutat 2019;40(2):142-161. (In eng). DOI: 10.1002/humu.23688.

5. Bandsma RH, van Goor H, Yourshaw M, et al. Loss of ADAM17 is associated with severe multiorgan dysfunction. Hum Pathol 2015;46(6):923-8. (In eng). DOI: 10.1016/j.humpath.2015.02.010.

6. Schlegel C, Lapierre LA, Weis VG, et al. Reversible deficits in apical transporter trafficking associated with deficiency in diacylglycerol acyltransferase. Traffic 2018;19(11):879-892. (In eng). DOI: 10.1111/tra.12608.

7. Burman A, Momoh M, Sampson L, et al. Modeling of a Novel Patient-Based MYO5B Point Mutation Reveals Insights Into MVID Pathogenesis. Cell Mol Gastroenterol Hepatol 2023;15(4):1022-1026. (In eng). DOI: 10.1016/j.jcmgh.2022.12.015.

8. Lee H, Huang AY, Wang LK, et al. Diagnostic utility of transcriptome sequencing for rare Mendelian diseases. Genet Med 2020;22(3):490-499. (In eng). DOI: 10.1038/s41436-019-0672-1.

9. Yourshaw M, Solorzano-Vargas RS, Pickett LA, et al. Exome sequencing finds a novel PCSK1 mutation in a child with generalized malabsorptive diarrhea and diabetes insipidus. J Pediatr Gastroenterol Nutr 2013;57(6):759-67. (In eng). DOI: 10.1097/MPG.0b013e3182a8ae6c.

10. Rockowitz S, LeCompte N, Carmack M, et al. Children's rare disease cohorts: an integrative research and clinical genomics initiative. NPJ Genom Med 2020;5:29. (In eng). DOI: 10.1038/s41525-020-0137-0.

11. Pedersen BS, Quinlan AR. Who's Who? Detecting and Resolving Sample Anomalies in Human DNA Sequencing Studies with Peddy. Am J Hum Genet 2017;100(3):406-413. (In eng). DOI: 10.1016/j.ajhg.2017.01.017.

12. Karczewski KJ, Francioli LC, Tiao G, et al. The mutational constraint spectrum quantified from variation in 141,456 humans. Nature 2020;581(7809):434-443. (In eng). DOI: 10.1038/s41586-020-2308-7.

13. Paila U, Chapman BA, Kirchner R, Quinlan AR. GEMINI: integrative exploration of genetic variation and genome annotations. PLoS Comput Biol 2013;9(7):e1003153. (In eng). DOI: 10.1371/journal.pcbi.1003153.

14. McKusick VA. Mendelian Inheritance in Man and its online version, OMIM. Am J Hum Genet 2007;80(4):588-604. (In eng). DOI: 10.1086/514346.

15. Rentzsch P, Witten D, Cooper GM, Shendure J, Kircher M. CADD: predicting the deleteriousness of variants throughout the human genome. Nucleic Acids Res 2019;47(D1):D886-D894. DOI: 10.1093/nar/gky1016.

16. Richards S, Aziz N, Bale S, et al. Standards and guidelines for the interpretation of sequence variants: a joint consensus recommendation of the American College of Medical Genetics and Genomics and the Association for Molecular Pathology. Genet Med 2015;17(5):405-24. DOI: 10.1038/gim.2015.30.

17. Lee H, Deignan JL, Dorrani N, et al. Clinical exome sequencing for genetic identification of rare Mendelian disorders. JAMA 2014;312(18):1880-7. (In eng). DOI: 10.1001/jama.2014.14604.

18. Coyaud E, Mis M, Laurent EM, et al. BioID-based Identification of Skp Cullin F-box (SCF)β-TrCP1/2 E3 Ligase Substrates. Mol Cell Proteomics 2015;14(7):1781-95. (In eng). DOI: 10.1074/mcp.M114.045658.

19. Kessner D, Chambers M, Burke R, Agus D, Mallick P. ProteoWizard: open source software for rapid proteomics tools development. Bioinformatics 2008;24(21):2534-6. (In eng). DOI: 10.1093/bioinformatics/btn323.

20. Craig R, Beavis RC. TANDEM: matching proteins with tandem mass spectra. Bioinformatics 2004;20(9):1466-7. (In eng). DOI: 10.1093/bioinformatics/bth092.

21. Eng JK, Jahan TA, Hoopmann MR. Comet: an open-source MS/MS sequence database search tool. Proteomics 2013;13(1):22-4. (In eng). DOI: 10.1002/pmic.201200439.

22. Liu G, Zhang J, Larsen B, et al. ProHits: integrated software for mass spectrometry-based interaction proteomics. Nat Biotechnol 2010;28(10):1015-7. (In eng). DOI: 10.1038/nbt1010-1015.

23. Teo G, Liu G, Zhang J, Nesvizhskii AI, Gingras AC, Choi H. SAINTexpress: improvements and additional features in Significance Analysis of INTeractome software. J Proteomics 2014;100:37-43. (In eng). DOI: 10.1016/j.jprot.2013.10.023.

24. Kiontke S, Langemeyer L, Kuhlee A, et al. Architecture and mechanism of the late endosomal Rab7-like Ypt7 guanine nucleotide exchange factor complex Mon1-Ccz1. Nat Commun 2017;8:14034. (In eng). DOI: 10.1038/ncomms14034.

25. Nelms B, Dalomba NF, Lencer W. A targeted RNAi screen identifies factors affecting diverse stages of receptor-mediated transcytosis. J Cell Biol 2017;216(2):511-525. (In eng). DOI: 10.1083/jcb.201609035.

26. Tyska MJ, Mooseker MS. MYO1A (brush border myosin I) dynamics in the brush border of LLC-PK1-CL4 cells. Biophys J 2002;82(4):1869-83. DOI: 10.1016/S0006-3495(02)75537-9.

27. Li SJ, Liu H, Wu FF, et al. Meshed neuronal mitochondrial networks empowered by AI-powered classifiers and immersive VR reconstruction. Front Neurosci 2023;17:1059965. (In eng). DOI: 10.3389/fnins.2023.1059965.

28. Suprynowicz FA, Krawczyk E, Hebert JD, et al. The human papillomavirus type 16 E5 oncoprotein inhibits epidermal growth factor trafficking independently of endosome acidification. J Virol 2010;84(20):10619-29. (In eng). DOI: 10.1128/JVI.00831-10.

29. Kroll F, Powell GT, Ghosh M, et al. A simple and effective F0 knockout method for rapid screening of behaviour and other complex phenotypes. Elife 2021;10 (In eng). DOI: 10.7554/eLife.59683.

30. Volpatti JR, Ghahramani-Seno MM, Mansat M, et al. X-linked myotubular myopathy is associated with epigenetic alterations and is ameliorated by HDAC inhibition. Acta Neuropathol 2022;144(3):537-563. (In eng). DOI: 10.1007/s00401-022-02468-7.

31. Ewels P, Magnusson M, Lundin S, Käller M. MultiQC: summarize analysis results for multiple tools and samples in a single report. Bioinformatics 2016;32(19):3047-8. (In eng). DOI: 10.1093/bioinformatics/btw354.

32. Bolger AM, Lohse M, Usadel B. Trimmomatic: a flexible trimmer for Illumina sequence data. Bioinformatics 2014;30(15):2114-20. (In eng). DOI: 10.1093/bioinformatics/btu170.

33. Kim D, Paggi JM, Park C, Bennett C, Salzberg SL. Graph-based genome alignment and genotyping with HISAT2 and HISAT-genotype. Nat Biotechnol 2019;37(8):907-915. (In eng). DOI: 10.1038/s41587-019-0201-4.

34. Danecek P, Bonfield JK, Liddle J, et al. Twelve years of SAMtools and BCFtools. Gigascience 2021;10(2) (In eng). DOI: 10.1093/gigascience/giab008.

35. Liao Y, Smyth GK, Shi W. featureCounts: an efficient general purpose program for assigning sequence reads to genomic features. Bioinformatics 2014;30(7):923-30. (In eng). DOI: 10.1093/bioinformatics/btt656.

36. Love MI, Huber W, Anders S. Moderated estimation of fold change and dispersion for RNA-seq data with DESeq2. Genome Biol 2014;15(12):550. (In eng). DOI: 10.1186/s13059-014-0550-8.

37. Durinck S, Spellman PT, Birney E, Huber W. Mapping identifiers for the integration of genomic datasets with the R/Bioconductor package biomaRt. Nat Protoc 2009;4(8):1184-91. (In eng). DOI: 10.1038/nprot.2009.97.

38. Thiagarajah JR, Kamin DS, Acra S, et al. Advances in Evaluation of Chronic Diarrhea in Infants. Gastroenterology 2018. DOI: 10.1053/j.gastro.2018.03.067.

39. Goulet O, Pigneur B, Charbit-Henrion F. Congenital enteropathies involving defects in enterocyte structure or differentiation. Best Pract Res Clin Gastroenterol 2022;56-57:101784. (In eng). DOI: 10.1016/j.bpg.2021.101784.

40. Overeem AW, Posovszky C, Rings EH, Giepmans BN, van IJzendoorn SC. The role of enterocyte defects in the pathogenesis of congenital diarrheal disorders. Dis Model Mech 2016;9(1):1-12. (In eng). DOI: 10.1242/dmm.022269.

41. Canani RB, Castaldo G, Bacchetta R, Martín MG, Goulet O. Congenital diarrhoeal disorders: advances in this evolving web of inherited enteropathies. Nat Rev Gastroenterol Hepatol 2015;12(5):293-302. (In eng). DOI: 10.1038/nrgastro.2015.44.

42. Babcock SJ, Flores-Marin D, Thiagarajah JR. The genetics of monogenic intestinal epithelial disorders. Hum Genet 2023;142(5):613-654. (In eng). DOI: 10.1007/s00439-022-02501-5.

43. Erickson RP, Larson-Thomé K, Valenzuela RK, Whitaker SE, Shub MD. Navajo microvillous inclusion disease is due to a mutation in MYO5B. Am J Med Genet A 2008;146A(24):3117-9. (In eng). DOI: 10.1002/ajmg.a.32605.

44. Salomon J, Espinosa-Parrilla Y, Goulet O, et al. A founder effect at the EPCAM locus in Congenital Tufting Enteropathy in the Arabic Gulf. Eur J Med Genet 2011;54(3):319-22. (In eng). DOI: 10.1016/j.ejmg.2011.01.009.

45. Haas JT, Winter HS, Lim E, et al. DGAT1 mutation is linked to a congenital diarrheal disorder. J Clin Invest 2012;122(12):4680-4. (In eng). DOI: 10.1172/JCI64873.
